# Atom-precise fluorescent copper cluster for tumor microenvironment targeting and transient chemodynamic cancer therapy

**DOI:** 10.1186/s12951-021-01207-6

**Published:** 2022-01-06

**Authors:** Zhenzhen Yang, Anli Yang, Wang Ma, Kai Ma, Ya-Kun Lv, Peng Peng, Shuang-Quan Zang, Bingjie Li

**Affiliations:** 1 Department of Oncology, The First Affiliated Hospital of Zhengzhou University, Zhengzhou, 450052 China; 2 Henan Key Laboratory of Crystalline Molecular Functional Materials, Henan International Joint Laboratory of Tumor Theranostical Cluster Materials, Green Catalysis Center, and College of Chemistry, Zhengzhou University, Zhengzhou, 450001 China; 3 Department of Breast Oncology, State Key Laboratory of Oncology in South China, Collaborative Innovation Center for Cancer Medicine, Sun Yat-sen University Cancer Center, Guangzhou, 510060 China

**Keywords:** Nano clusters, Sustainable release, Targeting property, ROS, Cancer therapy

## Abstract

**Background:**

Reactive oxygen species (ROS) have been widely studied for cancer therapy. Nevertheless, instability and aspecific damages to cellular biomolecules limit the application effect. Recently, significant research efforts have been witnessed in the flourishing area of metal nanoclusters (NCs) with atomically precise structures for targeted release of ROS but few achieved success towards targeting tumor microenvironment.

**Results:**

In this work, we reported an atomically precise nanocluster Cu_6_(C_4_H_3_N_2_S)_6_ (Cu_6_NC), which could slowly break and generate ROS once encountered with acidic. The as-prepared Cu_6_NC demonstrated high biological safety and efficient chemodynamic anti-tumor properties. Moreover, Cu_6_NC enabled transient release of ROS and contained targeting behavior led by the tumor microenvironment. Both in vitro and in vivo experiments confirmed that Cu_6_NC demonstrated a low cytotoxicity for normal cells, while presented high cytotoxicity for tumor cells with a concentration-dependent manner.

**Conclusions:**

This work not only reported a promising candidate for chemodynamic cancer therapy, but also paved the route to address clinical issues at the atomic level.

**Graphical Abstract:**

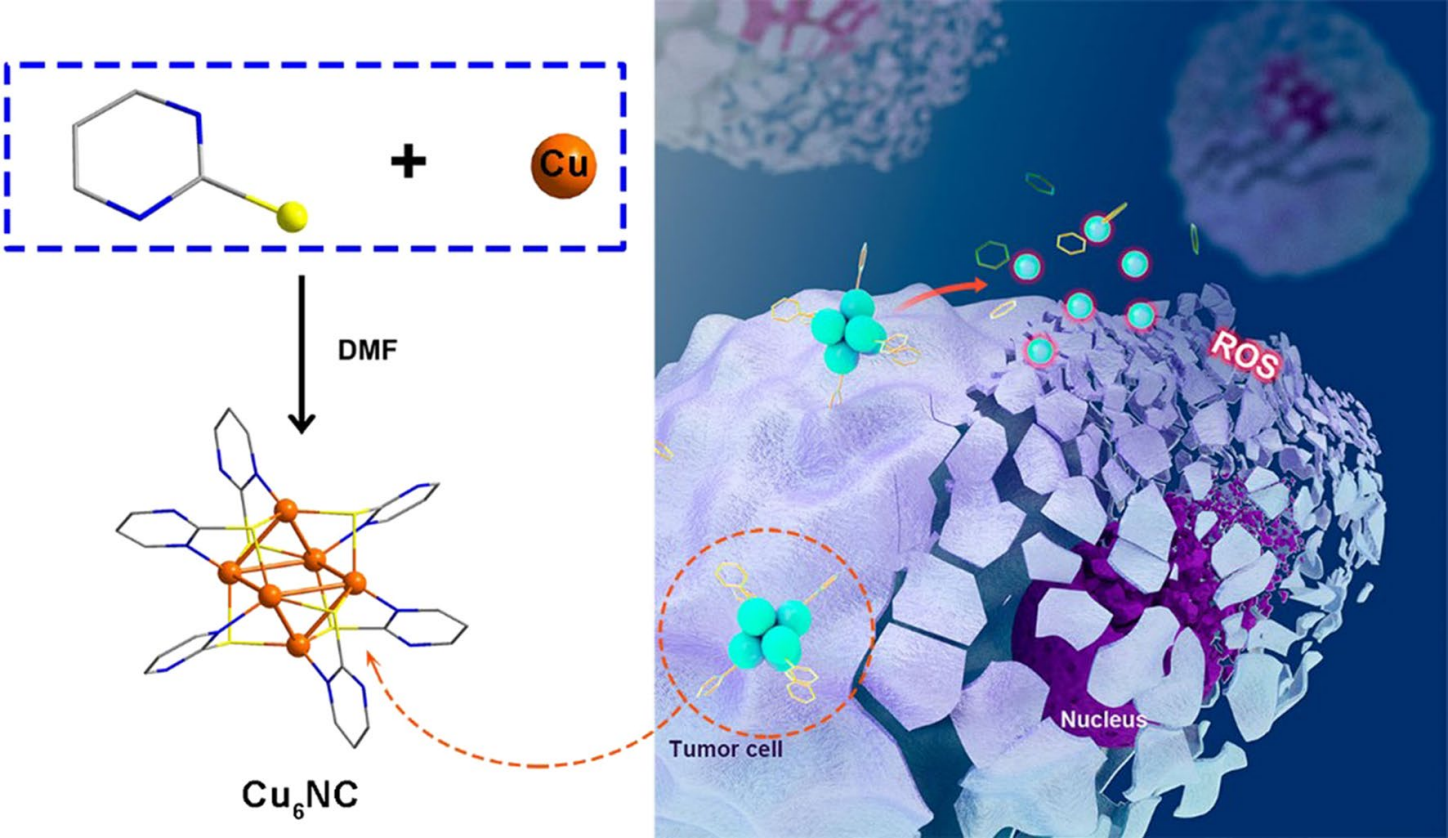

**Supplementary Information:**

The online version contains supplementary material available at 10.1186/s12951-021-01207-6.

## Introduction

Reactive oxygen species (ROS) are unstable molecules that contain oxygen, such as singlet oxygen, superoxide, hydroxyl radical and peroxide, etc. For the last decades, both experimental and clinical studies confirm that the alterations of ROS can affect the intracellular redox state [1–3]. Ever since, numerous efforts have been devoted to examine the role of ROS in cellular transformation and tumorigenesis [4, 5]. It has been observed that raising ROS level could effectively promote the apoptosis of cancer cells and modulate the immune response (specifically, inhibition) towards the treatment of cancer as well as autoimmune diseases [6–8]. Moreover, the elevation of ROS levels also contributes to tissue regeneration [9]. Many treatments such as the exposure of cancer cells to chemotherapy and radiotherapy could upregulate ROS [10–12]. Nevertheless, caused by the intrinsic instability, it is difficult for ROS to realize sustainable release for persistent anti-cancer effect. Meanwhile, a well-coordinated and balanced redox system is typically present under normal physiological conditions. Thus, promoting ROS may produce cellular stress and damage, since ROS can damage cellular biomolecules (such as proteins, DNA, RNA) and result in mutations or even carcinogenesis. Hence, the controllable presentation of ROS is vital to take advantage of this double-edged sword for cancer therapy.

Over the past decades, remarkable progress has been achieved in the area of nanomaterials such as atomically precise assembled nanoclusters [13–16]. Amongst the nanoclusters, atomically precise metal nanoclusters (NCs) has explored promising potentials with fine-tuned properties [17–19]. The bottom-up programmed assembly of these NCs make it relatively simple to control over the size and to replace the surface organic ligand, providing good opportunities for particular applications [20–24]. By far, various metal based NCs (especially gold) have demonstrated effective anti-cancer properties [25, 26]. For example, researchers have developed NCs that could make cancer cells more sensitive to radiation and promote the efficiency of radiotherapy [27–30]. Our previous work also explored photothermic behavior from coordinated-metal centers for cancer therapy [31]. By far, our group have designed and synthesized dozens of metal NCs with atomically precise assembly [32–38]. Benefiting from the precise formulas, both the structure and the physicochemical properties of atomically precise metal NCs are controllable [39, 40]. Thus, one could reasonably hypothesize that with rational design, the additional introduction of the organic ligand could modulate targeting properties while the functional modification may generate sustained releasement of NCs in particular position.

**Scheme 1:**
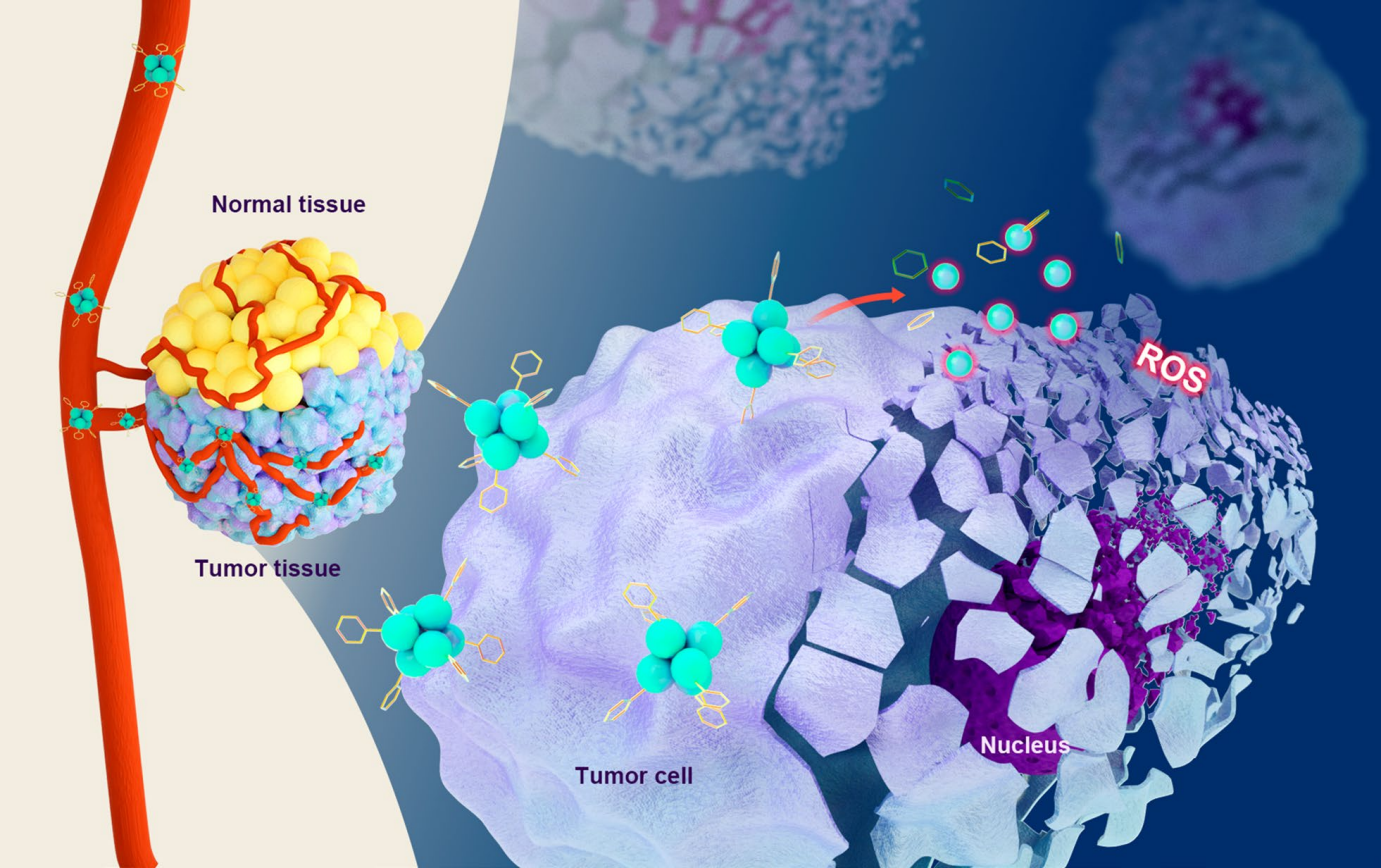
Schematic of the Cu_6_NC for chemodynamic anti-tumor therapy. Once encountered with the acidic tumor microenvironment, the Cu_6_NC would slowly break and give birth to ROS via a sustained releasement, leading to high cytotoxicity for tumor cells

Herein, we reported an atomically precise nanocluster Cu_6_(C_4_H_3_N_2_S)_6_, which were determined by the single crystal X-ray diffraction (SC-XRD). 2-mercaptopyrimidine was used to assemble the precise structures and led to a size of ~ 1 nm of each cluster as calculated from the SC-XRD analysis. Consistently, none of cardiotoxicity, kidney toxicity or liver toxicity were observed in the main organs and peripheral blood, suggesting the good compatibility of our Cu_6_NC. Specially, the structure Cu_6_(C_4_H_3_N_2_S)_6_ was quite stable unless mixed in acidic environment. The slight addition of acid (pH ~ 6) would slowly break the structure and generate the burst of radicals, realizing the sustained releasement of ROS (Scheme 1). The in vitro and in vivo experiments confirmed that Cu_6_NC demonstrated a low cytotoxicity for normal cells, while presented high cytotoxicity for tumor cells with a concentration-dependent manner (Scheme 1). Such observations not only confirmed the biological safety and chemodynamic anti-tumor properties, but also indicated the targeting behavior led by the tumor microenvironment.

## Results and discussions

### Synthesis and characterization of Cu_6_(C_4_H_3_N_2_S)_6_ nanoclusters

**Fig. 1:**
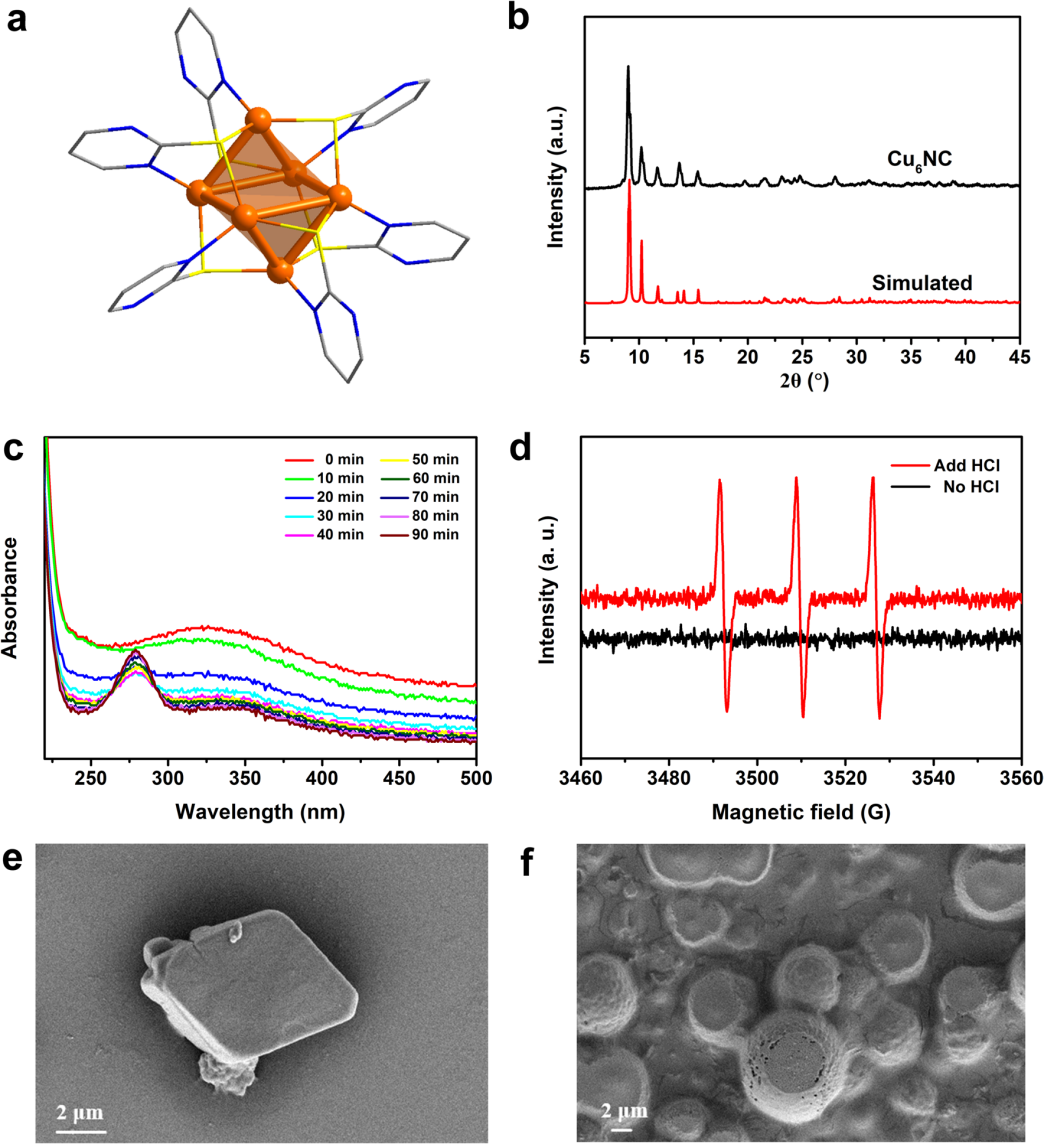
Characterization of Cu_6_NC. **a** The molecular structure of Cu_6_NC. **b** The experimental PXRD patterns and the simulated ones from the SC-XRD of Cu_6_NC, which were in good agreement. **c** The tracking monitoring of UV–vis absorption spectra for Cu_6_NC in HCl solution (pH ~ 6). **d** EPR of Cu_6_NC with and without the addition of HCl solution (pH ~ 6). The emerging peaks indicated the generation of ROS once HCl was added. **e** SEM images of the crystalline Cu_6_NC. Clearly shape could be observed. **f** SEM images of Cu_6_NC after the treatment of HCl solution (pH ~ 6). The crystals were totally pulverized

Typically, the Cu_6_(C_4_H_3_N_2_S)_6_ nanocluster (Cu_6_NC) was synthesized via one-pot route by blending 2-mercaptopyrimidine with Cu(NO_3_)_2_·3H_2_O (ratio of 1/1 by mole) in dimethyl formamide (detailed in “Experimental section”). According to the SC-XRD analysis, each of the ligand bridges three Cu ions. Specially, each sulfur atom of the ligand bridges two Cu ions, while each Cu ion is coordinated with two sulfur atoms and one nitrogen atom from three independent ligands (Fig. 1a). The exact structure information could be found in the Additional file 1: Table S1. As shown in Fig. 1b, the experimental Powder-XRD (PXRD) patterns were in good agreement with the simulated ones from the SC-XRD, indicating the phase purity of the as-synthesized crystalline products of Cu_6_NC. X-Ray photoelectron spectroscopy (XPS) (Additional file 1: Fig. S1a) confirmed that Cu_6_NC contained C, N, S and Cu elements. The peaks at 932.6 and 952.5 eV in the high-resolution Cu 2p spectrum (Additional file 1: Fig. S1b) could be attributed to the 2*p*_*2/3*_ and 2*p*_*1/2*_ of Cu^+^, respectively [41, 42], which was generated by the reductive mercapto groups. In the Fourier transform infrared (FTIR) spectra (Additional file 1: Fig. S2), the adsorption peaks of S–H stretch were absent in the spectra of Cu_6_NC and the C=C/C=N peaks were apparently red-shifted, suggesting the formation of the coordination between mercaptopyrimidine and Cu ions [43]. Such coordinated ligands led to considerable thermal stabilities. Under nitrogen atmosphere, the thermogravimetric analysis (TGA) revealed that large weight loss (~ 40%) was observed at 400 °C, suggesting that Cu_6_NC could be maintained from room temperature to nearly 400 °C (Additional file 1: Fig. S3).

Notably, Cu_6_NC demonstrated crucial luminescent properties, indicating potential applications for biomedical labeling and targeting. The normalized emission spectra of the Cu_6_NC showed long-wavelength emission bands of 700 nm (Additional file 1: Fig. S4), which was appealing for the biological test. Moreover, the fluorescence of Cu_6_NC was reserved in water as well as in many biological media (Additional file 1: Fig. S5), indicating that Cu_6_NC held considerable stability for the potential biological applications. According to dynamic light scattering (DLS) analysis, the average size of Cu_6_NC components in aqueous was ~ 23 nm (Additional file 1: Fig. S6), which was within the range enabling efficient uptake by tumors. Transmission electron microscopy (TEM) of the Cu_6_NC (Additional file 1: Fig. S7) demonstrated a homogenous distribution with the size around 20 nm, which was smaller than the kidney filtration threshold. Moreover, obvious light absorption of Cu_6_NC was observed at the wavelength of 370 nm during the solid state UV–*vis* absorption spectra test (Additional file 1: Fig. S8). Since the structure was quite stable in water, we further tracked the UV–*vis* absorption spectra curves of Cu_6_NC in the HCl solution (pH ~ 6), observing a slowly structure changes (Fig. 1c). Meanwhile, the PXRD revealed that the crystalline structures of Cu_6_NC were totally broken (Additional file 1: Fig. S9). More importantly, electron paramagnetic response (EPR) of Cu_6_NC displayed significantly changed signals with the addition of HCl solution (pH ~ 6) (Fig. 1d), proving that a large number of ROS was produced. On the contrary, none signals was detected when tested the pure ligands under the same condition (Additional file 1: Fig. S10). The morphology analysis by scanning electron microscopy (SEM) and the associated energy dispersive spectroscopy (EDS) also revealed that the crystalline Cu_6_NC with clear shape (Fig. 1e and Additional file 1: Fig. S11) was totally pulverized (Fig. 1f and Additional file 1: Fig. S12) after the treatment of acidic solution (pH ~ 6). This suggests that the slight addition of acid (pH ~ 6) would break the structure and generate the burst of radicals, realizing the releasement of ROS. We also performed the test of Fenton-like reactions with Cu_6_NC in different pH, and the result showed that Fenton-like reactions were stronger in lower pH conditions, supporting our results that Cu6NC can generate ROS under acid environment (Additional file 1: Fig. S13). The above results indicate that Cu_6_NC maintains a stable structure in the simulated neutral normal tissue environment (pH ~ 7.4), and at the same time, rapidly dissociates and releases the inner core and generates ROS after entering the simulated acidic tumor microenvironment (pH ~ 6).

### Cytotoxicity studies of Cu_6_NC

**Fig. 2:**
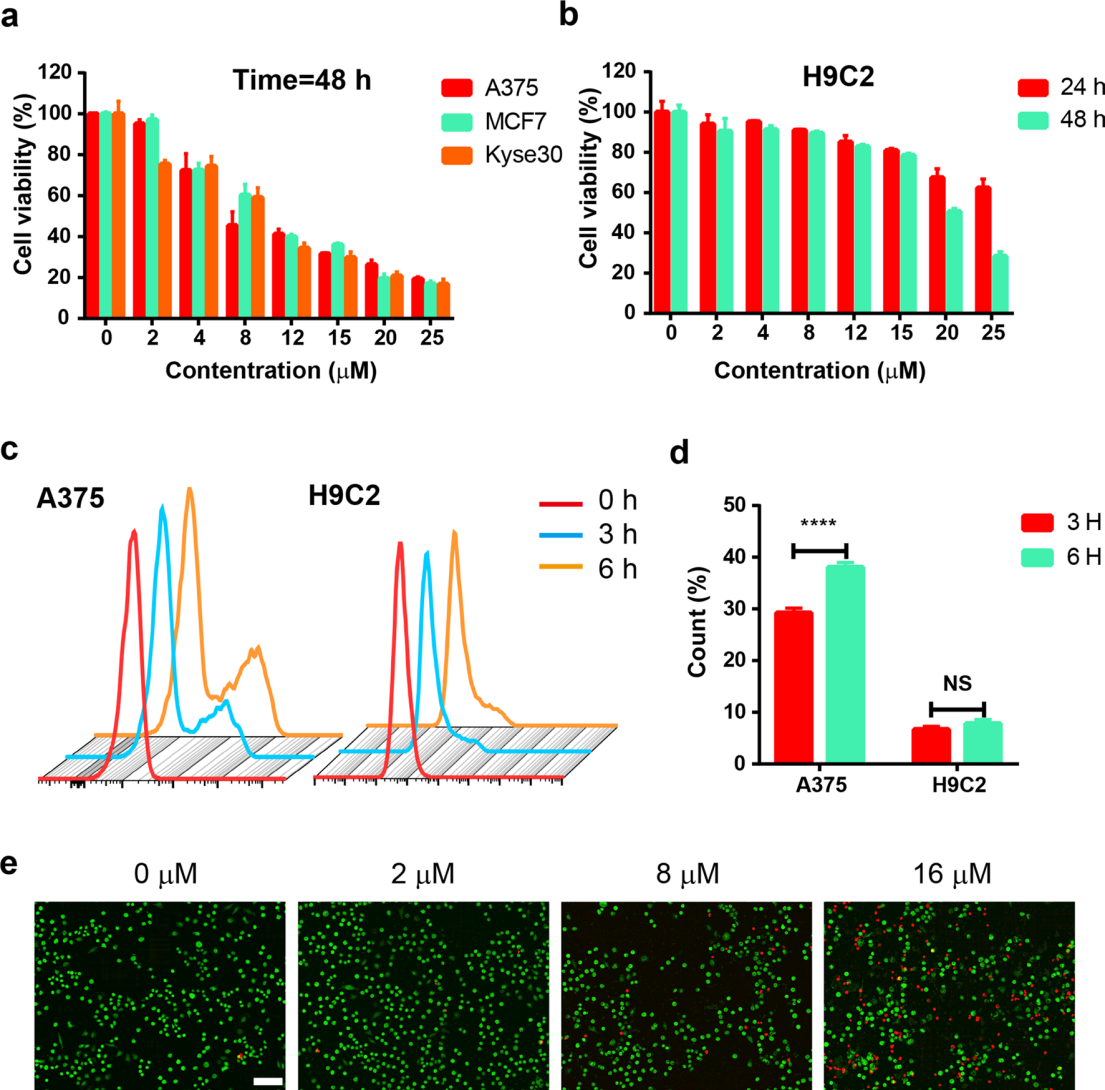
In vitro toxicity and cellular uptake of Cu_6_NC. **a** CCK-8 results of the viability of different cancer cells cultured with Cu_6_NC for 48 h. **b** CCK-8 results of the viability of H9C2 cells cultured with Cu_6_NC for 24 h and 48 h. **c** and** d** Flow cytometric results reflect the proportion of positive cells corresponding to the cellular uptake capacity of Cu_6_NC after the incubation of different durations for A375 cells and H9C2 cells. **e** Live/dead imaging of A375 cells after receiving different treatments. Scale bar: 50 μm

The biological safety of Cu_6_NC was evaluated by cell viability. Both normal (cardiomyocytes, H9C2) and cancer cell lines were used as models to assess cellular responses by CCK-8 assay kit. Cu_6_NC demonstrated high cytotoxicity to tumor cells (Additional file 1: Fig. S14 and Fig. 2a). After treatment with 20 μM Cu_6_NC for 48 h, the cell viability of A375, MCF-7 and Kyse30 cancer cell lines were 26.3%, 19.6%, and 20.9%, respectively (Fig. 2a). And its ligand components showed almost no effect on tumor cell viability (Additional file 1: Fig. S15). This is consistent with the EPR results (Fig. 1d and Additional file 1: Fig. S10), so we further confirmed the unique mechanism of Cu_6_NC leading to cytotoxicity through structural fragmentation. On the contrary, low cytotoxicity was observed in normal cardiomyocytes for Cu_6_NC (Fig. 2b). After 48 h of incubation, the inhibition rate of 20 μM Cu_6_NC was only 49.3% and the cell viability was 78.5% at the 15 μM (administration level), which was much higher than that tumor cells at the same level. As we all know, compared with normal cells, tumor cells survive through more glycolytic pathways, which leads to an acidic microenvironment [44, 45]. The acid sensitivity of Cu_6_NC is the main reason for its weak cytotoxicity to normal cells compared with tumor cells. Notably, we also measured the cellular uptake capacity of Cu_6_NC into both A375 cells and normal cardiomyocytes (Fig. 2c, d) during flow cytometric test. Along with the increase of incubation durations, the proportion of positive cells was slightly raised in cardiomyocytes while obviously promoted in cancer cells. We speculated that tumor cells could attract more Cu_6_NC during the instantaneous consumption of Cu_6_NC, and the less reaction of Cu_6_NC with cardiomyocytes determines the less cellular uptake. Therefore, we believed that the uptake of Cu_6_NC was more effective in tumor cells. These phenomena confirmed that Cu_6_NC was tumor-specific.

### Investigations of anti-tumor properties

**Fig. 3:**
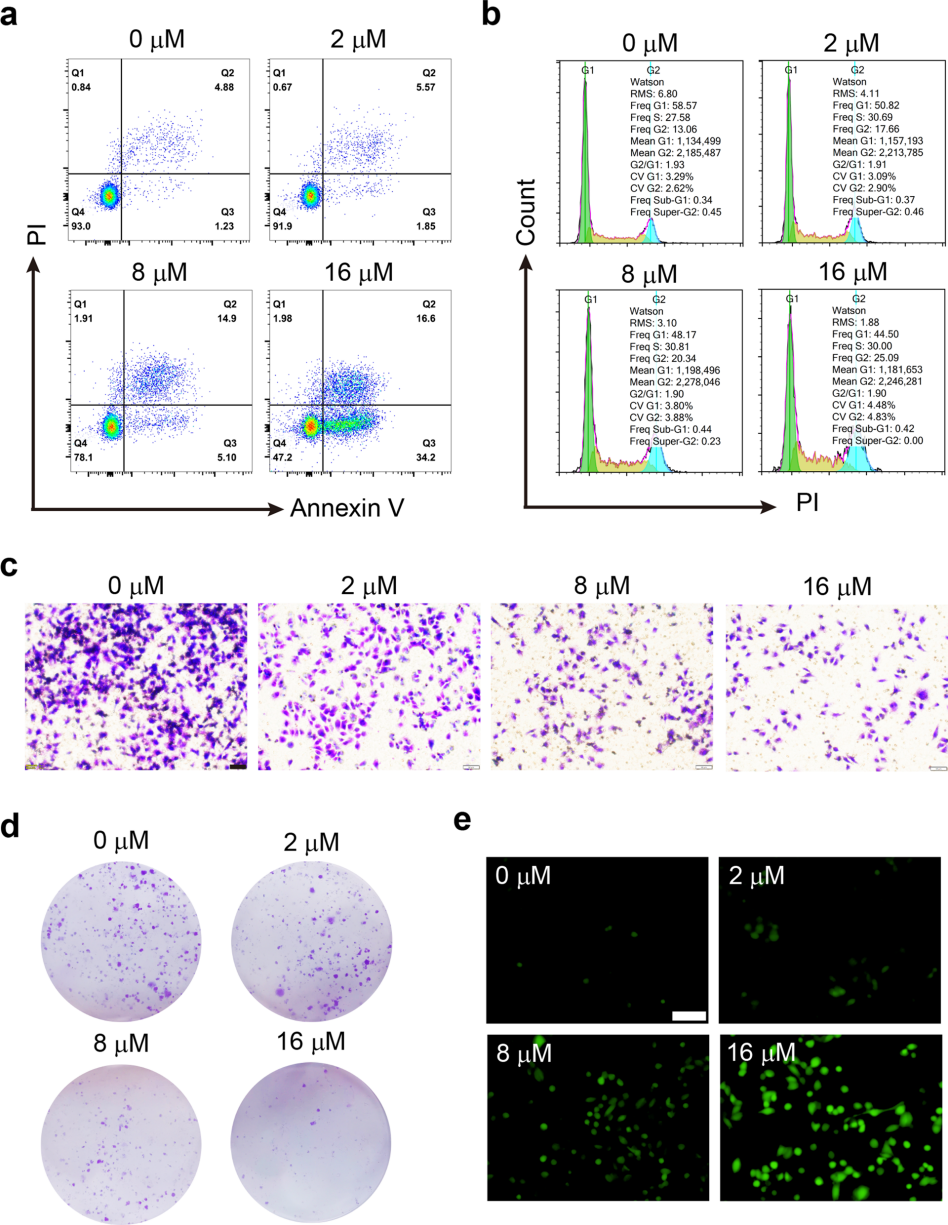
Chemokinetic anti-tumor properties of Cu_6_NC. Cell flow cytometry analysis for apoptosis. **a** and cell cycle **b** distribution in A375 cells after different concentrations (0, 2, 8 and16 μM) of Cu_6_NC treatment. **c** Representative images of transwell migration assay. Scale bar: 50 μm. **d** Typical images representing the colony formation ability of A375 cells with different treatments. **e** ROS production in A375 cells after Cu_6_NC treatment with different concentrations. Scale bar: 100 μm

Due to the selective cytotoxic property, Cu_6_NC was thoroughly evaluated for chemodynamic therapy (CDT) in vitro. The four groups of cells treated with different concentrations of Cu_6_NC (0, 2, 8 and 16 μM) were analyzed. Subsequently, the apoptosis and cell cycle were evaluated by flow cytometry after 24 h. As shown in Fig. 3a and Additional file 1: Fig. S16, Cu_6_NC promoted the apoptosis of A375 cells, thereby causing cell proliferation inhibition. Particularly, Cu_6_NC could mediate cell arrest in G2 phase (Fig. 3b and Additional file 1: Fig. S17). Live/dead stain imaging showed more red fluorescence appeared with the increased concentration of Cu_6_NC (Fig. 2e), suggesting that the viability of cells in the Cu_6_NC decreased in a dose-dependent manner. Meanwhile, live/dead detection associated with flow cytometry (Additional file 1: Figs. S18 and S19) also demonstrated that after incubation with 0, 2, 8 and 16 μM Cu_6_NC, the cell death rates were 3.63%, 7.14%, 23.0% and 42.2%, respectively. Since the formidable metastasis ability is one of the reasons that tumor cells are difficult to completely clean, we also performed transwell migration assay in vitro and observed that Cu_6_NC could significantly inhibit tumor cells migration (Fig. 3c and Additional file 1: Fig. S20). Furthermore, the cell colony formation assay was conducted to assess the chemodynamic efficacy of Cu_6_NC over a longer period of time. The corresponding results revealed similar dose-dependent (Fig. 3d and Additional file 1: Fig. S21). In the control group (0 μM), the colonies were densely packed and none effect on cell proliferation was observed. Specifically, the cell survival fraction obviously decreased with the addition of Cu_6_NC. In the 16 μM group, even almost no colonies appeared, indicating that Cu_6_NC was excellent for CDT.

Cu_6_NC induced considerable oxidative stress in vitro, leading to a specific tumor killing effect. Since ROS was detected during EPR test, we explored the underlying mechanisms of Cu_6_NC for CDT and detected the intracellular ROS by the probe DCFH-DA, which displayed green fluorescence upon oxidation by ROS. For A375 cells exposed to Cu_6_NC 12 h, the observed green fluorescence intensity was enhanced with the increasing of concentration (Fig. 3e and Additional file 1: Fig. S22), suggesting production of ROS via Cu^+^-mediated Fenton reaction. In addition, by detecting the production of ROS in A375 cells at different time points (Additional file 1: Fig. S23), it was shown that Cu_6_NC could slowly and sustainably release ROS for at least 48 h. Moreover, we found that the ratio of GSH/GSSG in A375 cells treated with Cu_6_NC for 6 h was significantly reduced in a concentration-dependent manner (Additional file 1: Fig. S24), which also indicated that the higher intracellular GSH levels in tumor cells could also be one of the possible mechanisms.

As a transition metal element and also an essential trace element for human, copper has been widely researched for its chemodynamic potential in cancer therapy [46]. Since tumor microenvironment has weakly acidic pH, low oxygen, and high glutathione, once chemodynamic properties enter such environment, chemodynamic cancer therapy is activated via its interaction with glutathione and reinforced by H_2_O_2_ through release of ROS [47]. Cu_6_NC synthesized in this work is an ideal property for chemodynamic cancer therapy. On one hand, the materials characterization results showed that Cu_6_NC triggers Fenton-like reaction and generates ROS which the standard mechanism of CDT, indicating its chemodynamic performance to explain its cytotoxicity to cancer cells over normal cells. On the other hand, our biomedical assay reveals that cancer cells treated with Cu_6_NC were observed with a higher level of ROS generation and lower GSH/GSSG ratio, showing that chemodynamic cancer therapy is a mechanism of Cu_6_NC to treat cancer cells. Therefore, our results confirmed that Cu_6_NC holds a chemodynamic property with potential for the treatment of cancer.

### In vivo cancer therapy

**Fig. 4:**
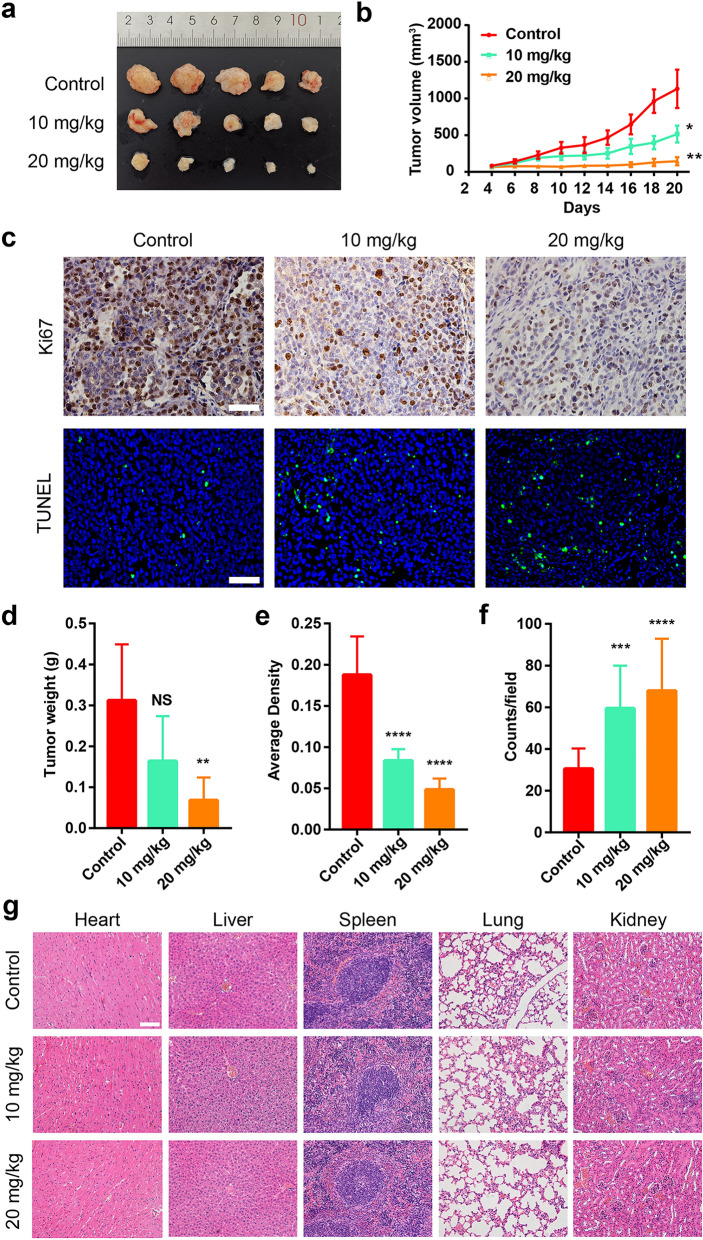
In vivo cancer chemotherapy results of Cu_6_NC. All mice were randomly divided into 3 groups: normal control group (Control), Cu_6_NC group (10 mg/kg), and Cu_6_NC group (20 mg/kg), at the same time, were treated by intraperitoneal injection of the corresponding dose of Cu_6_NC. **a** Photographs of mice after 16 days of different formulations. **b** Tumor growth curves of mice in different treatment groups. **c** Ki67 staining and TUNEL staining of tumor sections from different treatment groups. Scale bar: 100 μm. **d** Comparison of tumor weight in mice after therapy. The quantitative analysis of Ki67 **e** and TUNEL **f** performed by Image J software. **g** Pathological analysis of various organs in mice injected with different formulations. Scale bar: 50 μm

Inspired by the good in vitro CDT efficacy, tumor formation assay was performed in vivo by using human melanoma A375 tumor-bearing mice model. Mice with tumors of about 100 mm^3^ were randomly divided into three groups: (1) Control group, (2) Cu_6_NC group (10 mg/kg), and (3) Cu_6_NC group (20 mg/kg). According to the corresponding dose of each group, Cu_6_NC was administered intraperitoneally every 2 days. The tumor volume was measured every other day (Fig. 4a, b). At the time of final harvest, the tumor volume in the control group increased rapidly, while in the dose of 20 mg/kg group the tumor volume was markedly reduced by about 4.5 times (compared with the control group). Besides, the measured tumor weight also decreased significantly with the increase of the treatment dose (Fig. 4d). To verify the inhibitory effect of Cu_6_NC, the proliferation and apoptosis in tumor tissues from each group were further detected. According to the immunohistochemical staining, the Ki67 protein level of the Cu_6_NC treatment group was significantly lower than that of the control group (Fig. 4c, e). Consistently, the TUNEL staining also demonstrated a promotion with the increase of the administration concentration (Fig. 4c, f), further proving the ability of Cu_6_NC to induce apoptosis.

To evaluate the in vivo biocompatibility of Cu_6_NC, the main organs and peripheral blood of the mice were investigated after the mice were sacrificed. Specifically, H&E staining results of the mice in every group showed that no major pathological abnormalities was induced in main organs of mice (Fig. 4g). Concomitantly, none of cardiotoxicity, kidney toxicity or liver toxicity from Cu_6_NC was observed in the blood samples (Additional file 1: Fig. S25), indicating the low in vivo toxicity of Cu_6_NC.

Therefore, the results confirmed that the mechanism of Cu_6_NC for cancer therapy includes CDT, where the break of Cu_6_NC generate ROS which plays a role in killing cancer cells [48]. Unlike many previous copper nanoparticles for CDT use where their structure is not atomically precise or with continuous release of ROS, Cu_6_NC demonstrates its strong strength in further clinical application due to its unusual chemodynamic mechanism [49, 50]. Firstly, Cu_6_NC is with atomically precise structure that its detection, application and possible side effects once for clinical use can be precisely examined, where there is not uncertain components or allotropic substances existing which may cause unpredictable danger. Secondly, Cu_6_NC does not break to generate ROS under environment of normal tissues and is with minor harm to normal tissues which has been tested both in vitro and in vivo, which guaranteed its bio-safety where normal tissues and important organs are not damaged heavily. More importantly, the generation of ROS requires Cu_6_NC to interact with tumor microenvironment and break, which means it only works when getting close to cancer cells. Since Cu_6_NC is made of copper element which is an essential trace element and a ligand which has been tested to be with high biocompatibility, it breaks to unharmful components after its chemodynamic cancer therapy with cancer cells which further minimize the potential danger in clinical use.

## Conclusions

ROS are unstable oxygen containing species, which make it difficult to be persistently subsistent and effective in clinical treatment. On the other hand, lacking of natural targeted properties, the surplus ROS are harmful to normal cells, generating damages or carcinogenesis. In this study, we designed and prepared Cu_6_NC with atomically precisely structures of Cu_6_(C_4_H_3_N_2_S)_6_, which could generate large amount of ROS in acidic environment. Such characters address the above conundrum: (1) Cu_6_NC could slowly degrade and sustainably release ROS; (2) the fragmentation of Cu_6_NC are induced under acidic environment, providing targeted properties. Thus, Cu_6_NC could be fragmented under the acidic tumor microenvironment with sustainable burst of ROS. Both in vitro and in vivo experiments confirm that Cu_6_NC not only present high cytotoxicity to tumor cells, but also ensure the biological safety. Our intelligent strategy not only develops a promising candidate for the targeted CDT of cancer cells, but also paves the way for addressing clinical issues at the atomic level.

## Experimental section

### Synthesis of Cu_6_(C_4_H_3_N_2_S)_6_

2-Mercaptopyrimidine (≥ 99%) was purchased from Alfa Aesar. N,N-dimethylformamide (DMF) and Copper nitrate trihydrate(II) (Cu(NO)_3_, ≥ 99%) were bought from Sinopharm Chemical Reagent Co., Ltd., China. All reagents and solvents used were of commercially available reagent grade and used without further purification. 2-Mercaptopyrimidine (112 mg, 1 mmol) and Cu(NO_3_)_2_·3H_2_O (240 mg, 1 mmol) were dissolved in 20 ml DMF solution respectively, stir thoroughly to dissolve completely. Subsequently, add the DMF solution of 2-Mercaptopyrimidine dropwise to the solvent of Cu(NO_3_)_2_·3H_2_O. Cu_6_L_6_ powder crystals (C_24_H_18_Cu_6_N_12_S_6_) were obtained by filtration after vigorous stirring at room temperature for 2 h (a single crystal suitable for SCXRD measurements can be obtained by reducing the dosage in the same proportion).

### Characterization

Powder X-ray diffraction (PXRD) patterns of the samples were acquired on a Riguku D/Max-2500PC X-ray diffractometer with Cu radiation (λ = 1.54178 Å). Fourier transform infrared (FT-IR) spectroscopy was conducted using a Bruker ALPHA II FT-IR spectrometer. X-ray photoelectron spectroscopy (XPS) analysis was obtained on an ESCALAB 250 instrument operated at 150 W and 200 eV with mono chromated Al Kα radiation. Thermogravimetric analyses (TGA) were performed on an SDT 2960 thermal analyzer from room temperature (RT) to 800 °C at a heating rate of 10 °C/min under a nitrogen atmosphere. Dynamic light scattering (DLS) data were obtained by Horiba nano Partica SZ-100V2. UV–*vis* absorption spectra were recorded using a Hitachi UH4150 UV–visible spectrophotometer in the range of 200–700 nm. Emission and excitation spectra at RT were recorded with an Edinburgh FLS 1000 fluorescence spectrometer, and luminescence microscopy images were recorded on an Olympus BX53 microscope. Electron paramagnetic resonance (EPR) spectra were recorded by a Bruker A 300 EPR spectrometer.

### Single-crystal X-ray diffraction (SCXRD) analysis

SCXRD was performed on a Rigaku XtaLAB Pro diffractometer Cu-Kα radiation (λ = 1.54184 Å) at 200 K. Data collection and reduction were conducted with CrysAlisPro software. The structures were solved with intrinsic phasing methods (SHELXT-2015) and refined by full-matrix least-squares on F2 using OLEX2, which utilizes the SHELXL-2015 module. The imposed restraints in least-squares refinement of each structure were commented in the corresponding CIF files. All non-hydrogen atoms were refined anisotropically, and the hydrogen atoms were included in idealized positions. The crystal structures are visualized by DIAMOND 3.2. The detailed information of the crystal data, data collection and refinement results are summarized in Additional file 1: Table S1.

### Fenton-like reaction

The colorimetric method was used to analyze the absorbance change of methylene blue (MB), indicating that ⋅OH is produced. Solutions of 25 mg/L MB, 10 mM H_2_O_2_, and 0.1 mM Cu_6_NC were formed at different pH values. After 2 h of incubation, the color changes of the solution were obtained.

### Cell culture

Cell lines including human melanoma cells (A375), human esophageal cancer cells (Kyse30), human breast cancer cells (MCF-7) and Rat cardiomyocytes cells (H9C2) were purchased from the cell bank of the Chinese Academy of Sciences (Shanghai, China). A375 and H9C2 cells were cultured in DMEM medium containing 10% FBS and 1% penicillin–streptomycin. MCF-7 and Kyse30 cells incubated in 1640 medium containing 10% FBS and 1% penicillin–streptomycin.

### Cytotoxicity analysis

A375, MCF-7 and Kyse30 cells were seeded in 96-well plates (8 × 10^3^ cells per well), respectively, and cultured for overnight. Then these cells were incubated with different concentrations of Cu_6_NC (0, 2, 4, 8, 12, 15, 20 and 25 μM), the incubation time was 24 h and 48 h, respectively. Finally, add 10 μL of CCK-8 solution to each well and incubate for another 1 h. The absorbance of cells in each well at 450 nm was measured by a microplate reader. The cytotoxicity test of Cu_6_NC on H9C2 cells was also carried out according to the above description. Correspondingly, the toxicity of the pure ligand to A375 cells was tested with CCK-8.

### Cellular uptake of Cu_6_NC

A375 and H9C2 cells were seeded in culture dishes and incubated for 24 h. Subsequently, cells were incubated with 8 μM Cu_6_NC and harvested after 3 h and 6 h. The percentage of positive cells was tested by the flow cytometry (ACEA NovoCyte3130, USA) with an excitation wavelength of 405 nm and an emission wavelength of 780 nm. Data were processed by FlowJo software.

### Apoptosis and cell cycle analysis

A375 (2.5 × 10^5^ cells per well) cells were seeded in 6-well plates with 0, 2, 8 and 16 μM Cu_6_NC for 24 h. The cells were collected using trypsin–EDTA and stained with annexin V-FITC/PI (BD Biosciences, USA) for 15 min, and then apoptotic cells were identified by flow cytometry. The cell cycle was measured by PI staining using a cell cycle analysis kit (KeyGen Biotech, China). After staining following the manufacturer’s instructions, the cells were analyzed by flow cytometry (FACS Aria III, USA). Data were processed by ModFit LT software.

### Cell dead/live assay

A375 cells were treated with Cu_6_NC (0, 2, 8 and 16 μM) for 24 h. Before being observed with a fluorescence microscope, the cells were washed twice with PBS and incubated with 2 μM calcein AM and 4.5 μM PI for 20 min. For flow cytometry detection, after being digested and harvested, the cells are washed twice by PBS and then tested by a FACS Aria III flow cytometer. Data were processed and analyzed by FlowJo software.

### Transwell migration assay

Migration assays of A375 in vitro were performed using a 24-well plate with a transwell polycarbonate permeable support (pore size, 8 μm; Corning Incorporated, Corning, USA). A375 cells were incubated and treated as described in the Apoptosis Assay. The harvested cells were resuspended in FBS-free medium and seeded in the upper chamber with 1 × 10^5^ cells per well. The lower chamber was filled with 20% FBS medium (600 μL/well). The cells were then cultured for 48 h. Finally, the cells in the lower layer of the upper chambers were fixed with 4% paraformaldehyde and stained with 0.1% crystal violet. Numbers of migrated cells were photographed with a fluorescence microscopy and processed by ImageJ software.

### Colony formation ability identification

A375 cells were incubated and treated as described in the Apoptosis Assay. The cells were digested and seeded in 6-well plates (1000 cells per well). Then the cells are cultured in complete medium, and the medium was changed every 4 days. At 12 days, the cells were fixed with 4% paraformaldehyde and stained with 0.1% crystal violet. The cell colonies were observed and photographed with a microscope, and the colonies containing more than 50 cells were counted using ImageJ software.

### Intracellular reactive oxygen species (ROS) generation

A375 cells (5 × 10^4^ cells/well) were incubated in a 24-well plate with different concentrations of Cu_6_NC (0, 2, 8 and16 μM) for 12 h. Then, the level of cellular ROS was measured by 2′,7-dichlorodihydrofluorescein diacetate (DCFH-DA) probe (Beyotime Biotechnology, Jiangsu, China). Firstly, the cells were washed twice with PBS, incubated with DCFH-DA (10 μM) for 25 min, and then washed with PBS three times. Finally, the ROS signal of the cells was observed by a fluorescence microscopy. In addition, according to the above method, the production of ROS in A375 cells after 8 μM Cu_6_NC treatment was detected at different times (0, 2, 4, 8, 12, 24, 36 and 48 h).

### Assessment of glutathione (GSH)

Levels of GSH in A375 cells after treatment with Cu_6_NC were evaluated with GSH and GSSG assay kits (Beyotime Biotechnology, Jiangsu, China). In short, A375 cells were seeded in 6-well dishes (2 × 10^5^ cells per well) and treated with different concentrations of Cu_6_NC (0, 2, 8 and 16 μM) for 6 h. Then, the cells were collected with centrifugation, and the supernatant was discarded. Samples were added protein remover M (10 mg/30 μL), subjected to 3 cycles of freezing–thawing, and then centrifugated at 1000*g* for 10 min at 4 °C. The supernatant was reserved for GSH measurement with GSH and GSSG assay kits according to the manufacturer’s protocol.

### In vivo chemodynamic therapy

All animal procedures were conducted in accordance with the Guide for the Care and Use of Laboratory Animals and were approved by the Welfare and Ethics Review Committee of Zhengzhou University Laboratory Animal Center (Approval number: ZZU-LAC20200911[14]). In order to generate a xenograft mouse model, on day 0, we planted A375 cells (5 × 10^6^ tumor cells in 200 μL of PBS) in 6-week-old female BALB/c nude mice (Beijing Vital River Laboratory Animal Technology Co. Ltd, China) subcutaneously in the right flank. When the tumor volume reached about 100 mm^3^ on the 4th day, we randomly divided the mice into three groups: normal control group (Control), Cu_6_NC group (10 mg/kg), and Cu_6_NC group (20 mg/kg), at the same time by intraperitoneal injection corresponding dose of Cu_6_NC to treat mice. Mice were exposed to these treatments every other day for a total of nine administrations. The length (L) and width (W) of the tumor were measured with a vernier caliper every 2 days, and the tumor volume (V) was calculated using the formula: V = L × W^2^/2. On the 20th day, the tumor tissues, peripheral blood, and major organs (heart, liver, spleen, lungs and kidneys) of mice were harvested.

All harvested tissues were fixed with 4% formalin at least for 48 h. The fixed tissues were used for experiments such as hematoxylin and eosin (H&E) and immunohistochemistry staining. Apoptosis of tumor tissue sections was detected by staining with terminal deoxynucleotidyl transferase dUTP nick end labeling (TUNEL). The proliferation of tumor tissue was detected by immunohistochemical Ki67 staining. H&E were used to stain sections of major organs in each group. All these stained sections could be observed with a fluorescence microscopy. After the peripheral blood was collected, a serum sample was obtained by centrifugation. Then, an automatic biochemical analyzer (Chemray 240 or 840, Rayto, China) was used to detect the indexes of liver function, kidney function and myocardial enzymes.

### Statistical analysis

All results are presented as the mean ± standard deviation (SD). Two treatment groups were compared by Student’s t test. Multiple group comparisons were performed by two-way analysis of variance with Tukey’s post hoc test. All statistical analyses were carried out using GraphPad Prism 5. *p < 0.05, **p < 0.01, ***p < 0.001 and ****p < 0.0001 were deemed as significant differences. “NS” indicates no significant differences.

### Supplementary Information

**Additional file 1.** Additional table and figures.

## Data Availability

The datasets supporting the conclusions of this article are included within the article and its additional file.
