# Correction: Atom-precise fluorescent copper cluster for tumor microenvironment targeting and transient chemodynamic cancer therapy

**DOI:** 10.1186/s12951-024-02691-2

**Published:** 2024-07-31

**Authors:** Zhenzhen Yang, Anli Yang, Wang Ma, Kai Ma, Ya-Kun Lv, Peng Peng, Shuang-Quan Zang, Bingjie Li

**Affiliations:** 1 Department of Oncology, The First Affiliated Hospital of Zhengzhou University, Zhengzhou, 450052 China; 2 Henan Key Laboratory of Crystalline Molecular Functional Materials, Henan International Joint Laboratory of Tumor Theranostical Cluster Materials, Green Catalysis Center, and College of Chemistry, Zhengzhou University, Zhengzhou, 450001 China; 3 Department of Breast Oncology, State Key Laboratory of Oncology in South China, Collaborative Innovation Center for Cancer Medicine, Sun Yat-sen University Cancer Center, Guangzhou, 510060 China

**Correction: Journal of Nanobiotechnology (2022) 20:20** 10.1186/s12951-021-01207-6

In this article the wrong figure appeared as Fig. 4. The kidney data was inadvertently duplicated from liver by mistake, and the data for 20 mg/kg group of heart was inadvertently duplicated from 10 mg/kg group in Fig. 4g. The correction of this information does not affect the results and conclusions of this paper. The corrected image of Fig. 4 is provided below.

Uncorrected figure
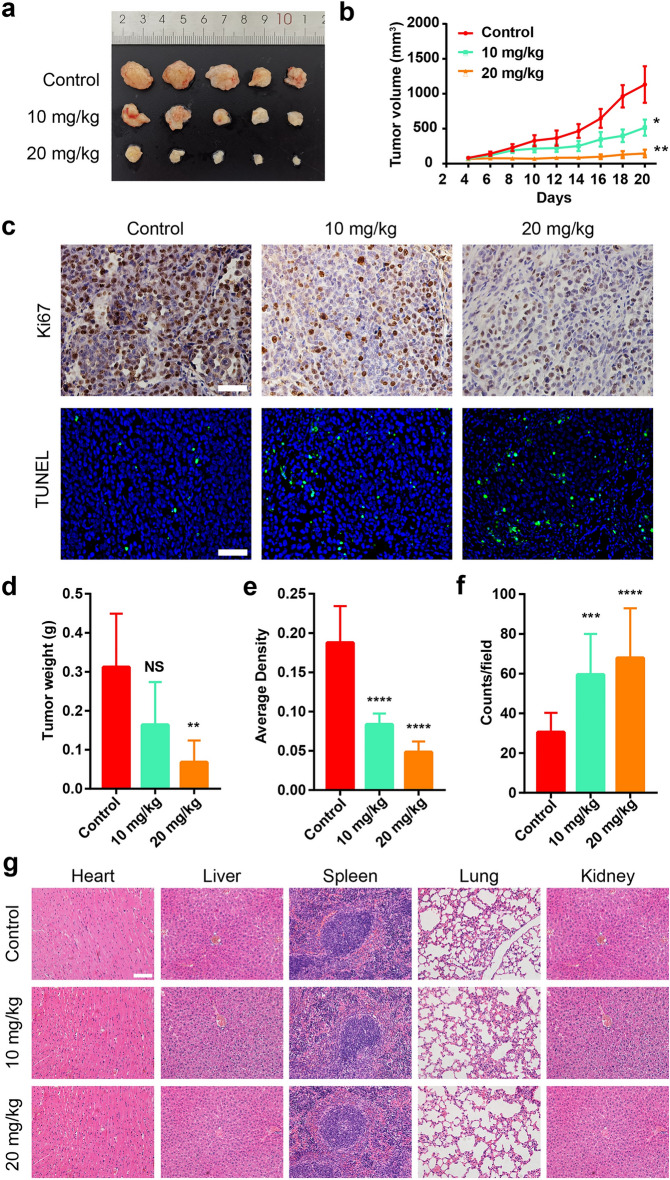


Corrected figure

**Fig. 4:**
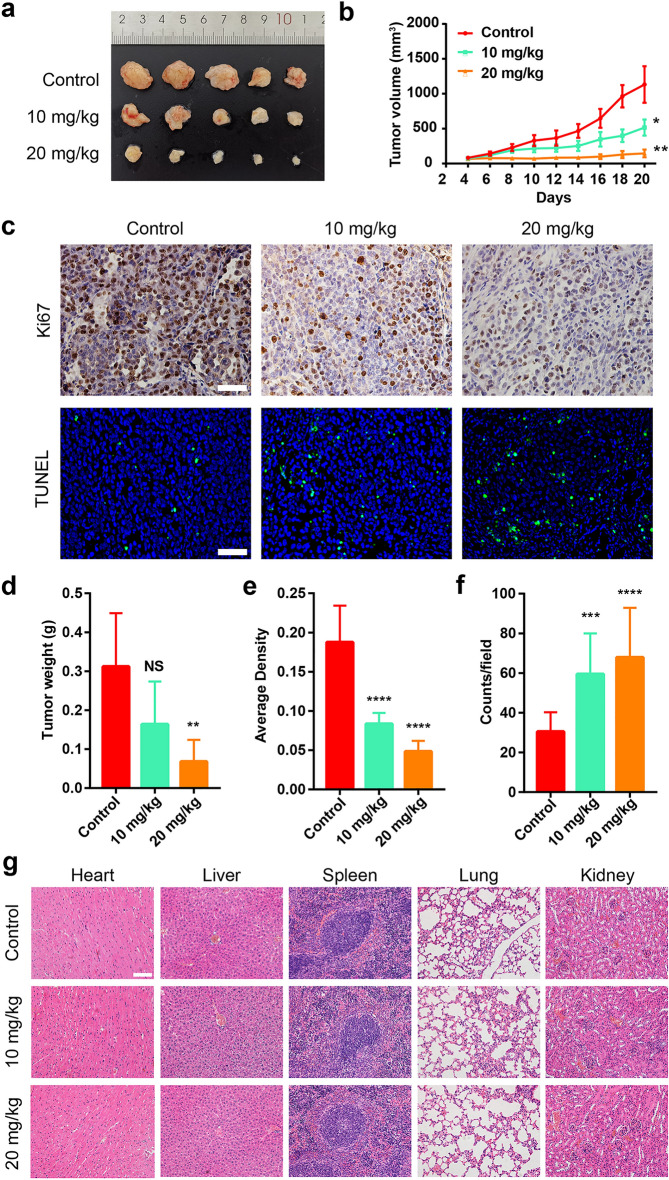
In vivo cancer chemotherapy results of Cu_6_NC. All mice were randomly divided into 3 groups: normal control group (Control), Cu_6_NC group (10 mg/kg), and Cu_6_NC (20 mg/kg), at the same time, were treated by intraperitoneal injection of the corresponding dose of Cu_6_NC. **a** Photographs of mice after 16 days of different formulations. **b** Tumor growth curves of mice in different treatment groups. **c** Ki67 staining and TUNEL staining of tumor sections from different treatment groups. Scale bar: 100 μm. **d** Comparison of tumor weight in mice after therapy. The quantitative analysis of Ki67 **e** and TUNEL **f** performed by Image J software. **g** Pathological analysis of various organs in mice injected with different formulations. Scale bar: 50 μm

The original article has been corrected.

