# Supplementary material for: Atom-precise fluorescent copper cluster for tumor microenvironment targeting and transient chemodynamic cancer therapy
Source: J Nanobiotechnology. 2022 Jan 6;20:20. doi: 10.1186/s12951-021-01207-6 (PMC8734230; doi:10.1186/s12951-021-01207-6)
Supplement: Supplementary file 1 — Additional file 1. Additional table and figures. [file 12951_2021_1207_MOESM1_ESM.docx]

Additional Information

Atom-precise fluorescent copper cluster for tumor microenvironment targeting and transient chemodynamic cancer therapy

Zhenzhen Yang ^1, #^, Anli Yang^3, #^, Wang Ma^1^, Kai Ma^2^, Ya-Kun Lv^2^, Peng Peng^1, 2, *^, Shuang-Quan Zang^2,*^, Bingjie Li^1, 2,*^

*^1^* Department of Oncology, The First Affiliated Hospital of Zhengzhou University, Zhengzhou 450052, China

*^2^* Henan Key Laboratory of Crystalline Molecular Functional Materials, Henan International Joint Laboratory of Tumor Theranostical Cluster Materials, Green Catalysis Center, and College of Chemistry, Zhengzhou University, Zhengzhou 450001, China

*^3^* Department of Breast Oncology, State Key Laboratory of Oncology in South China, Collaborative Innovation Center for Cancer Medicine, Sun Yat-sen University Cancer Center, Guangzhou 510060, China

^#^These authors contributed equally to the work.

*^*^*Corresponding authors.

E-mail: [ppeng@zzu.edu.cn](mailto:ppeng@zzu.edu.cn); [zangsqzg@zzu.edu.cn](mailto:zangsqzg@zzu.edu.cn); [bingjieli1991@outlook.com](mailto:bingjieli1991@outlook.com)

**Additional Tables**

**Table S1.** Crystal data and structure refinement information for Cu_6_NC.

| **Compound** | **Cu_6_NC** |
| --- | --- |
| CCDC number | 2083549 |
| Empirical formula | \|  \| C_30_H_32_Cu_6_N_14_O_2_S_6_ \| \| --- \| --- \| |
| Formula weight | 1194.29 |
| Temperature / K | 199.99(10) |
| Crystal system | triclinic |
| Space group | P-1 |
| *a* /Å | 10.0702(2) |
| *b* /Å | 11.7425(2) |
| *c* /Å | 17.9207(3) |
| *α* /° | 90.4010(10) |
| *β* /° | 105.473(2) |
| *γ* /° | 92.479(2) |
| Volume /Å^3^ | 2040.02(7) |
| Z | 2.000 |
| *ρ*_calc_ g/cm^3^ | 1.944 |
| *μ* /mm^‑1^ | 6.705 |
| F(000) | 1192.0 |
| Crystal size/mm^3^ | 0.12 × 0.11 × 0.1 |
| Radiation | CuKα (λ = 1.54184) |
| 2Θ range for data collection /° | 5.118 to 148.216 |
| Index ranges | -12 ≤ *h* ≤ 12,-13 ≤ *k* ≤ 14,-11 ≤ *l* ≤ 22 |
| Reflections collected | 21566 |
| Independent reflections | 8008 [*R_int_* = 0.0337, *R_sigma_*= 0.0405] |
| Data/restraints/parameters | 8008/78/556 |
| Goodness-of-fit on F^2^ | 1.051 |
| Final *R* indexes [I>=2*σ* (I)] | *R_1_*= 0.0539, *wR_2_*= 0.1580 |
| Final *R* indexes [all data] | *R_1_*= 0.0628, *wR_2_*= 0.1662 |
| Largest diff. peak/hole / e Å^-3^ | 1.47/-0.75 |

**Additional Figures**


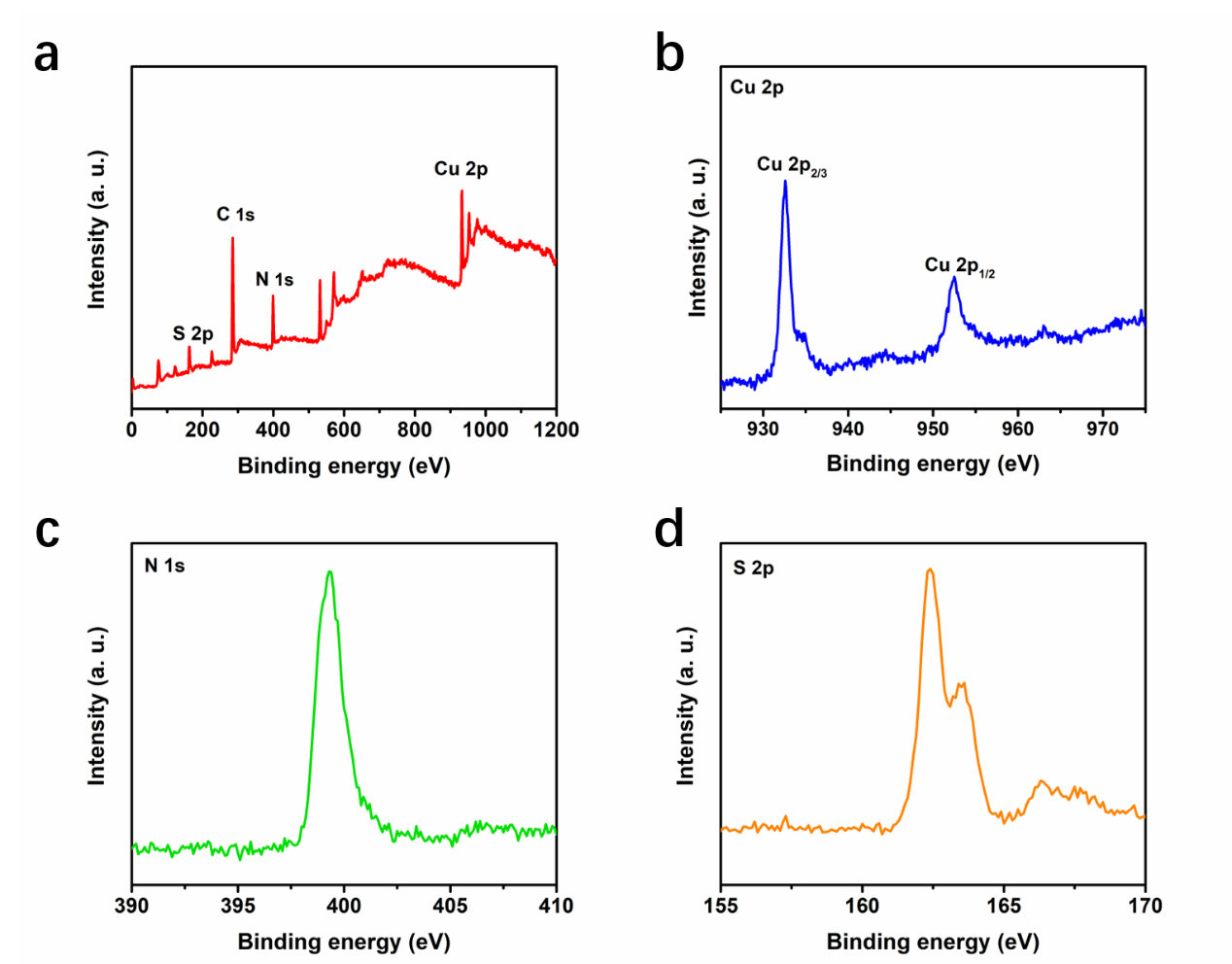


**Figure S1.** XPS analysis of Cu_6_NC. **a)** Survey of all elements. **b), c) and d)** High-resolution XPS for Cu 2p, N 1s and S 2p, respectively.





**Figure S2.** FTIR spectra of 2-mercaptopyrimidine (black line) and Cu_6_NC (red line).





**Figure S3.** TGA curves for Cu_6_NC. The first stage of the weight loss indicated the evaporation of the DMF, which was used as the solution during the synthesis.





**Figure S4.** Solid-state emission spectra of Cu_6_NC at 365 nm.





**Figure S5.** The fluorescence of Cu_6_NC in water and different biological media (1640, 1640+10%FBS, FBS, PBS, DMEM and DMEM+10%FBS). The peaks at ~700 nm indicated that Cu_6_NC was the well reserved.





**Figure S6.** DLS analysis of Cu_6_NC.


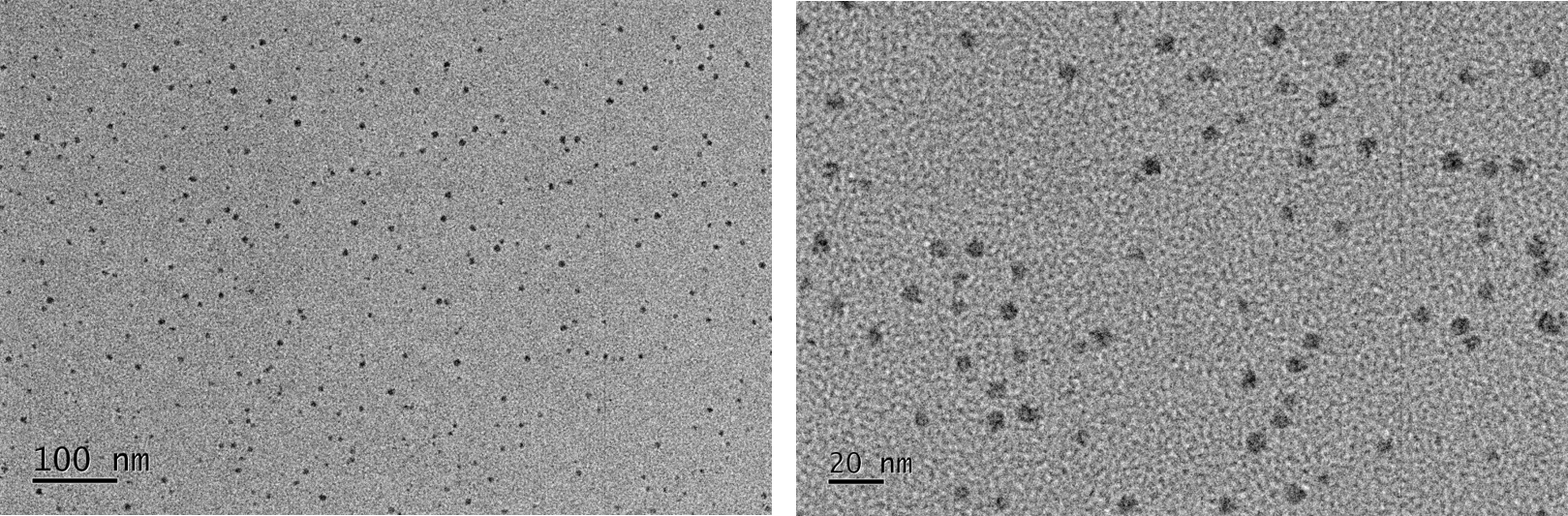


**Figure S7.** The TEM images of Cu_6_NC at different magnification. The clusters were aggregated and homogenously distributed.





**Figure S8.** Solid-state UV-*vis* absorption spectra of Cu_6_NC.





**Figure S9.** The PXRD patterns of Cu_6_NC before and after the treatment with HCl (pH~6).





**Figure S10.** EPR of pure ligand with and without the addition of HCl solution (pH~6). None signal was detected, indicating that ROS could not be generated.


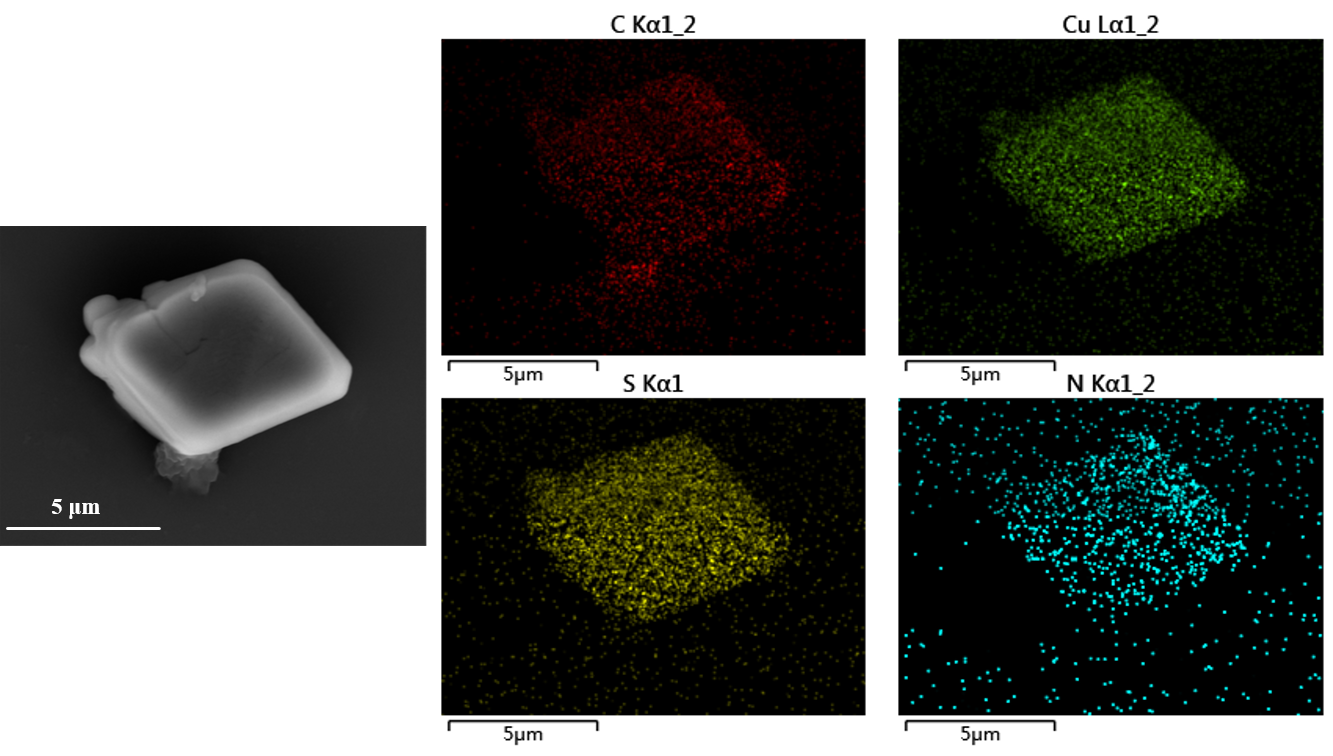


**Figure S11.** The SEM associated EDS images of crystalline Cu_6_NC. Cu, N, C and S elements were homogenously dispersed along the crystals.


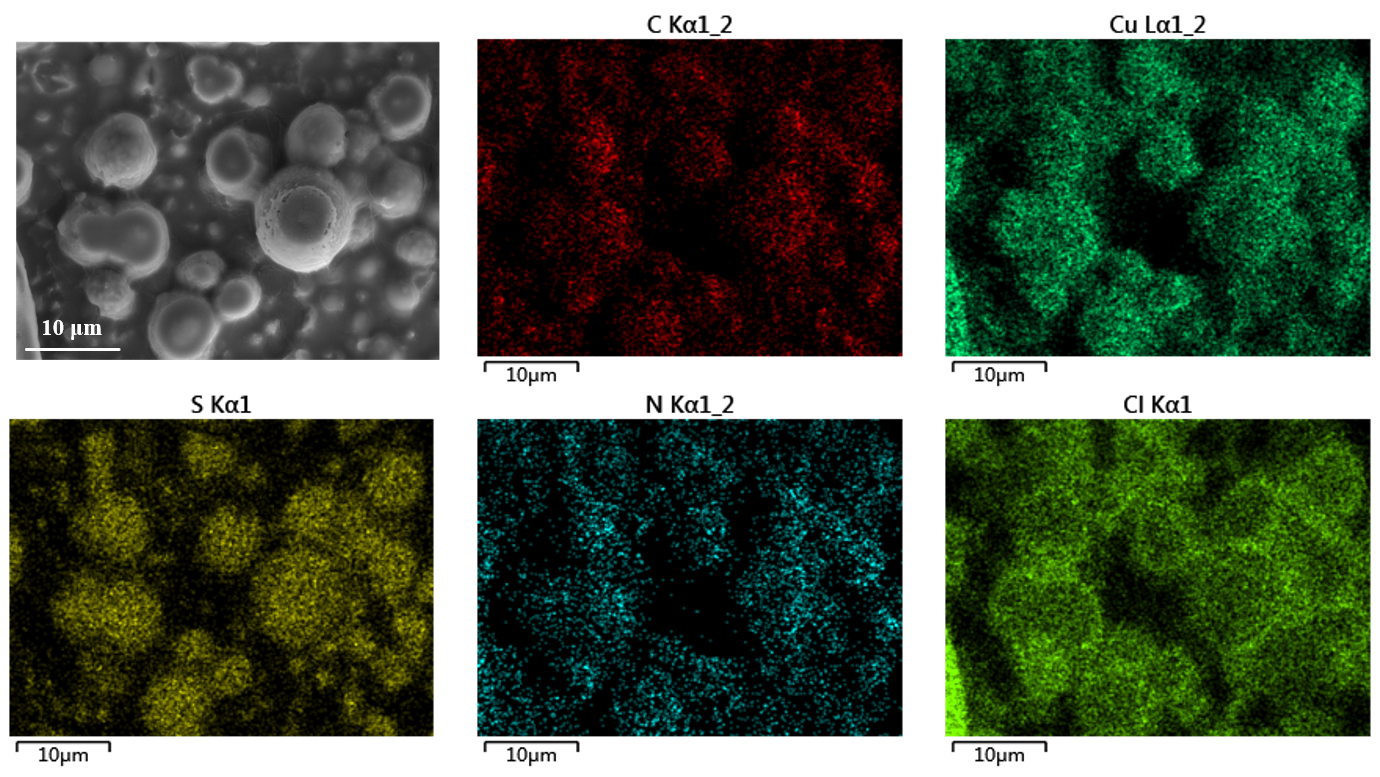


**Figure S12.** The SEM associated EDS images of the Cu_6_NC treated by acidic solution (pH~6). The Cu, N, C and S could still be observed with homogenous distribution, while the Cl was from the addition of HCl solution (pH~6). The crystals were totally pulverized.


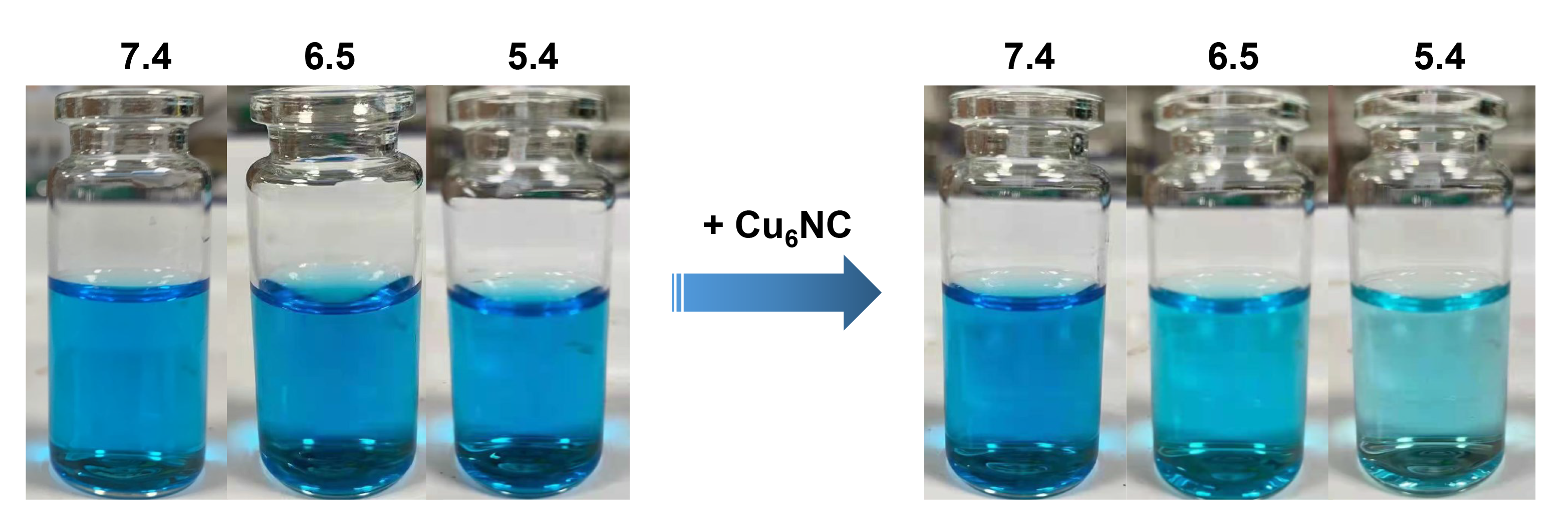


**Figure S13.** Images of MB solutions containing H_2_O_2_ at different pH’s values before and after adding Cu_6_NC, showing the Fenton-like reaction between Cu_6_NC and H_2_O_2_ to produce ·OH.


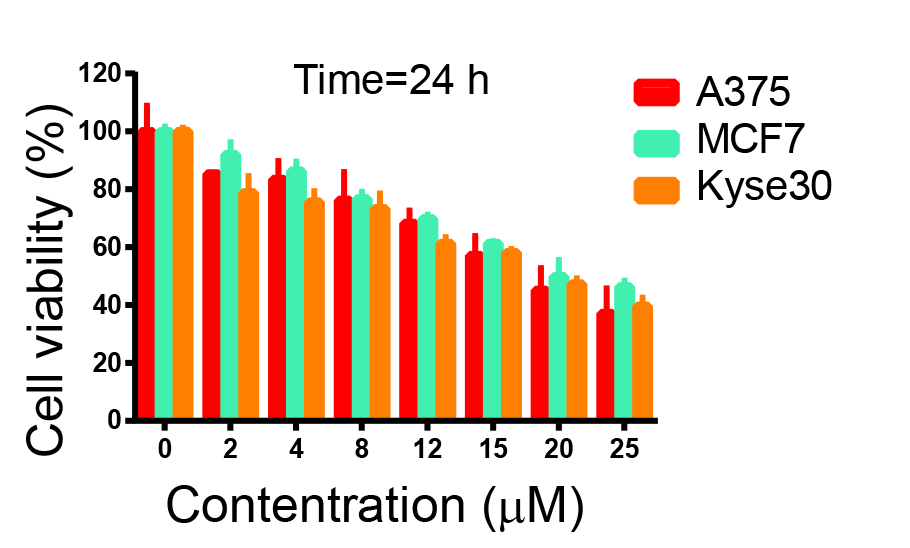


**Figure S14.** CCK-8 results of the viability of tumor cells cultured with Cu_6_NC for 24 h.


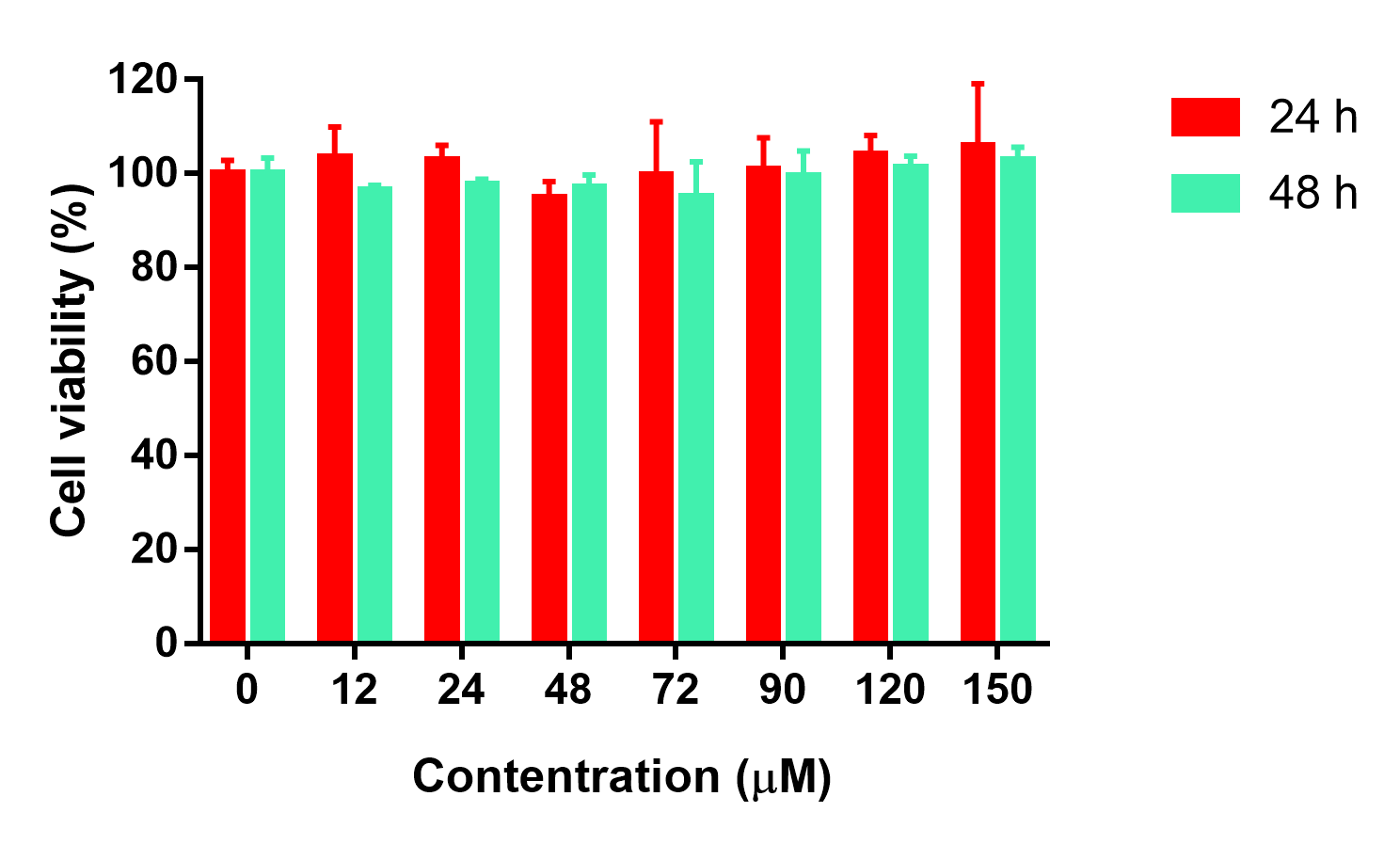


**Figure S15.** Cell viability of A375 cells cultured with different concentrations of pure ligand of Cu_6_NC for 24 hours and 48 hours.


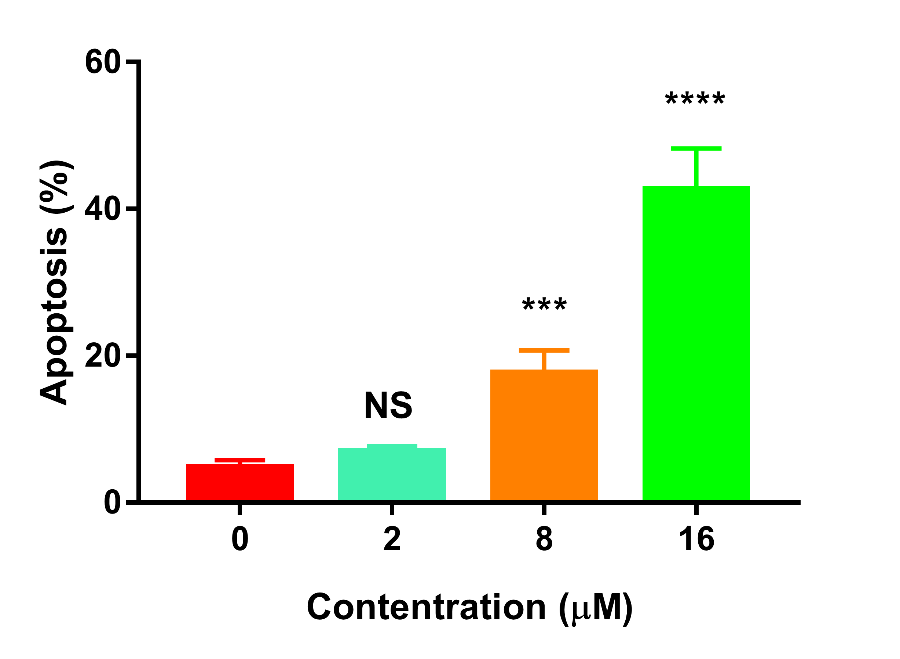


**Figure S16.** Statistical results of apoptosis ratio in A375 cells after different concentrations (0, 2, 8 and16 μM) of Cu_6_NC treatment.


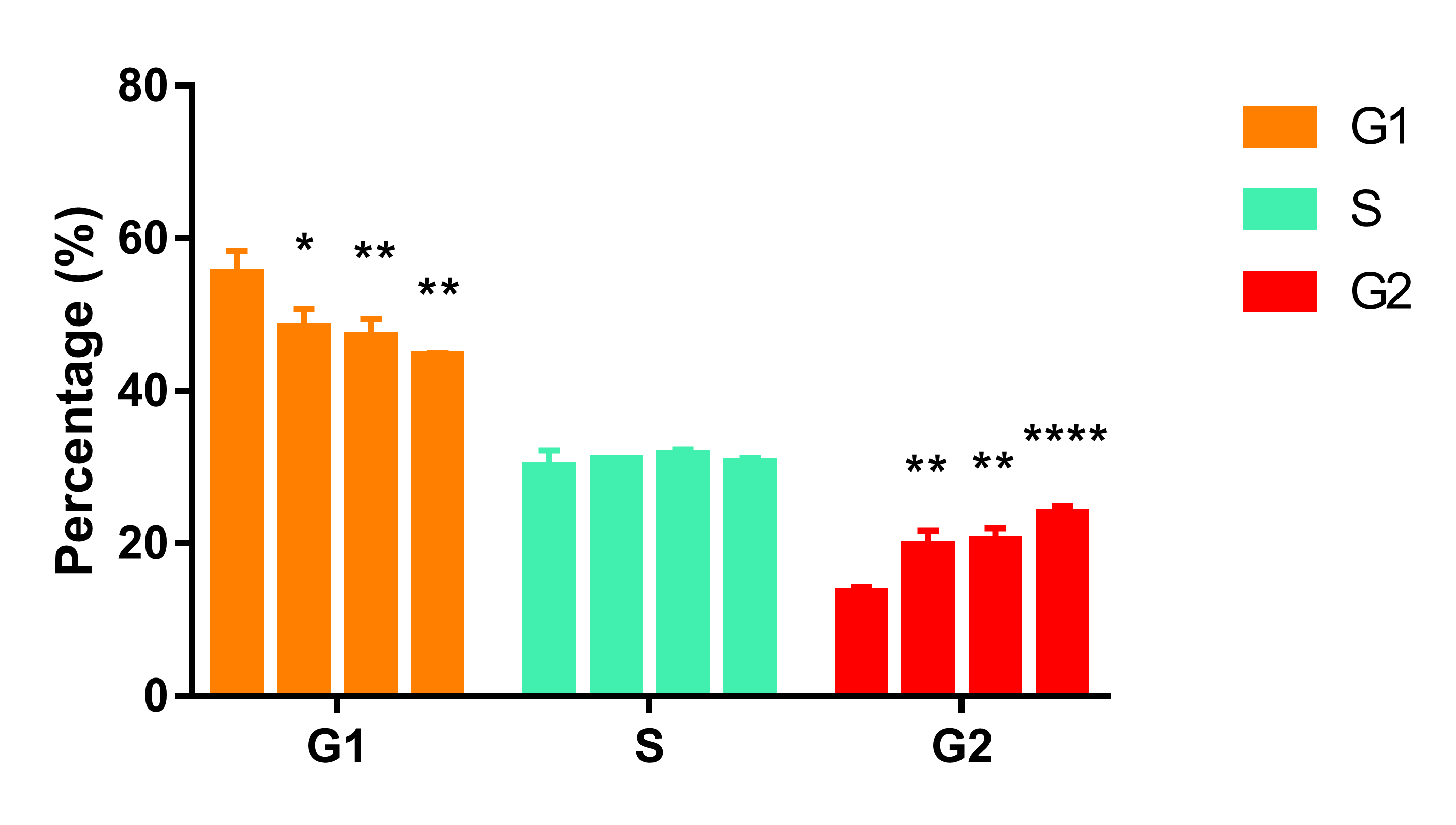


**Figure S17.** Statistical results of cell cycle in A375 cells after different concentrations (0, 2, 8 and 16 μM) of Cu_6_NC treatment.

**
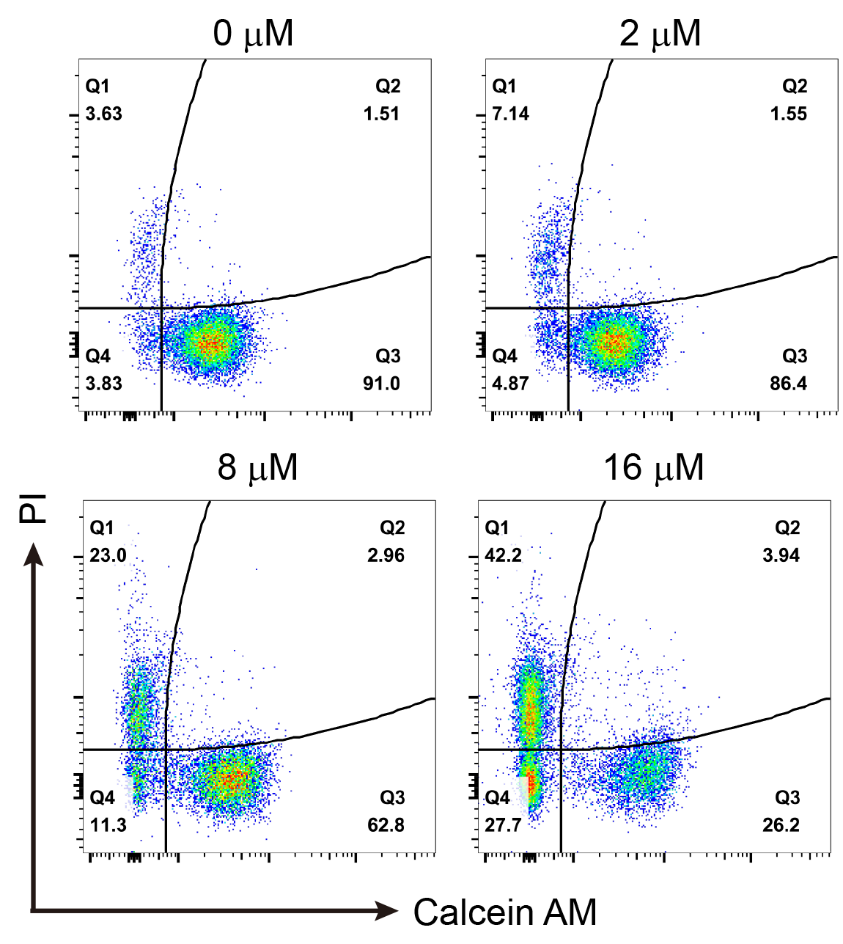
**

**Figure S18.** The live/dead status of A375 cells after different treatments detected by flow cytometry.


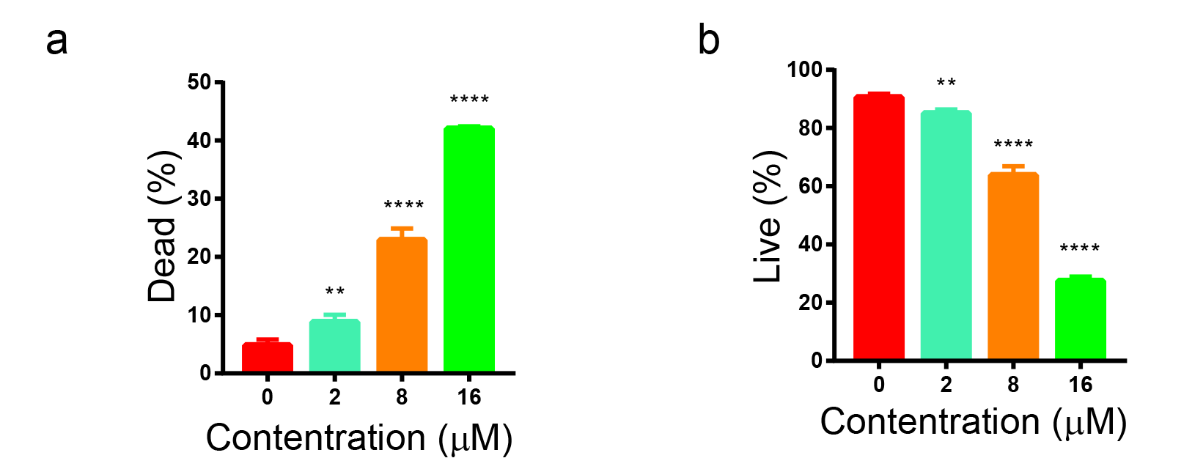


**Figure S19.** The live/dead status of A375 cells after different treatments analyzed by FlowJo software.


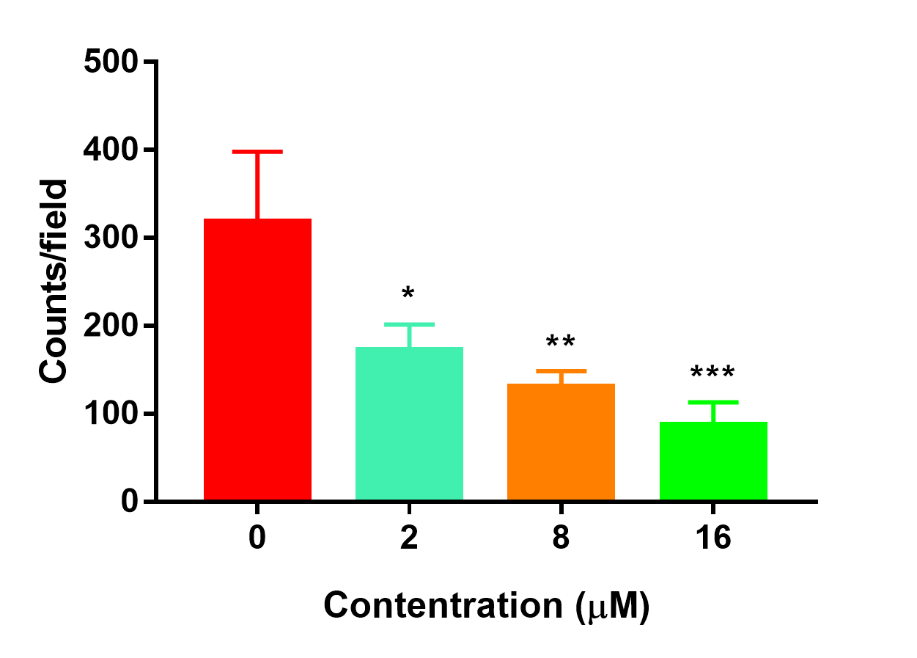


**Figure S20.** The statistical results of the migration number were counted with ImageJ software, after A375 cells were processed differently.


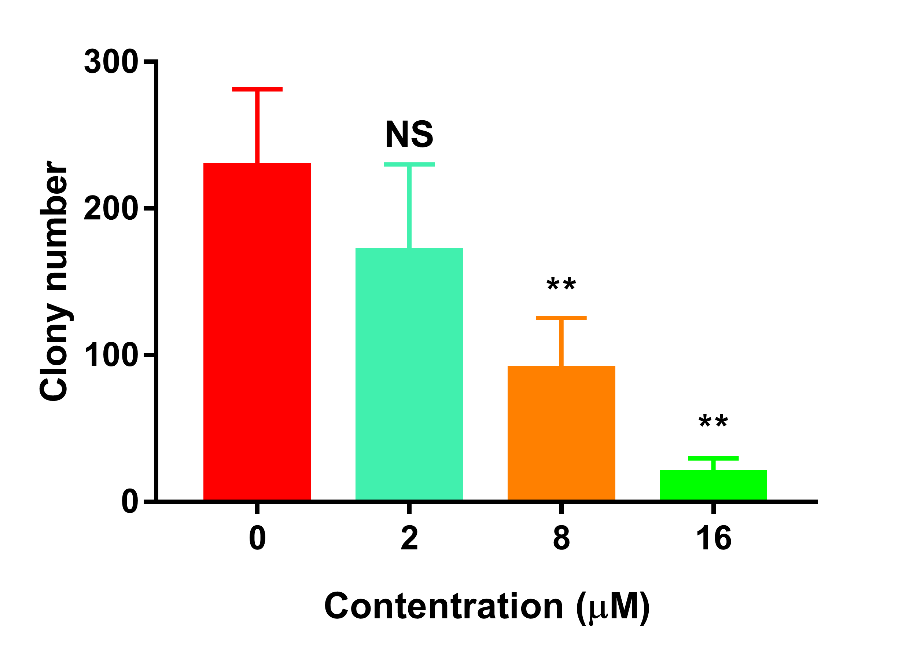


**Figure S21.** Quantitative analysis of the number of colonies for A375 cells undergo different treatments.


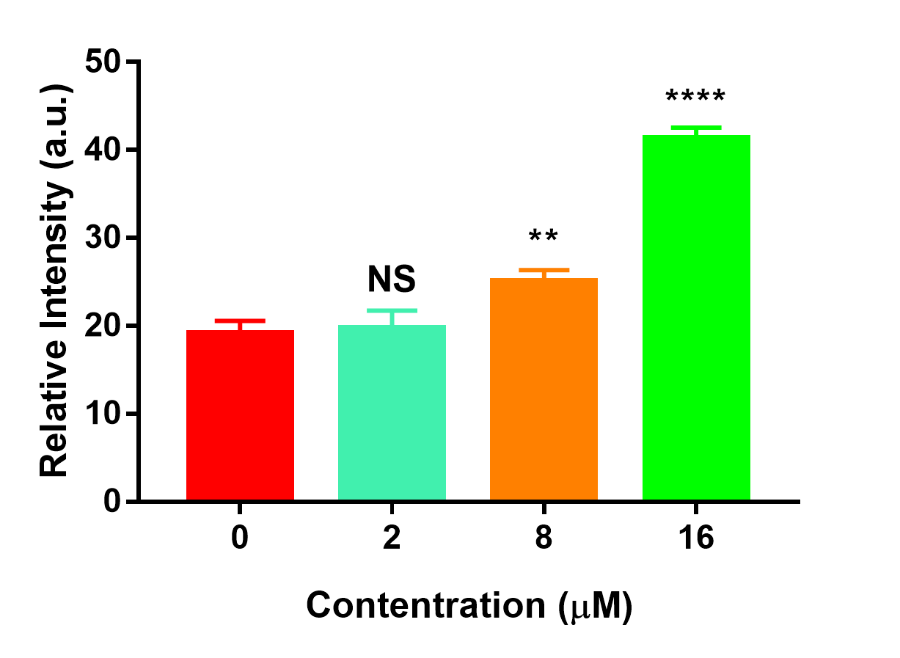


**Figure S22.** Quantitative analysis of ROS generation in A375 cells after Cu_6_NC treatment, detected by the DCFH-DA probe.


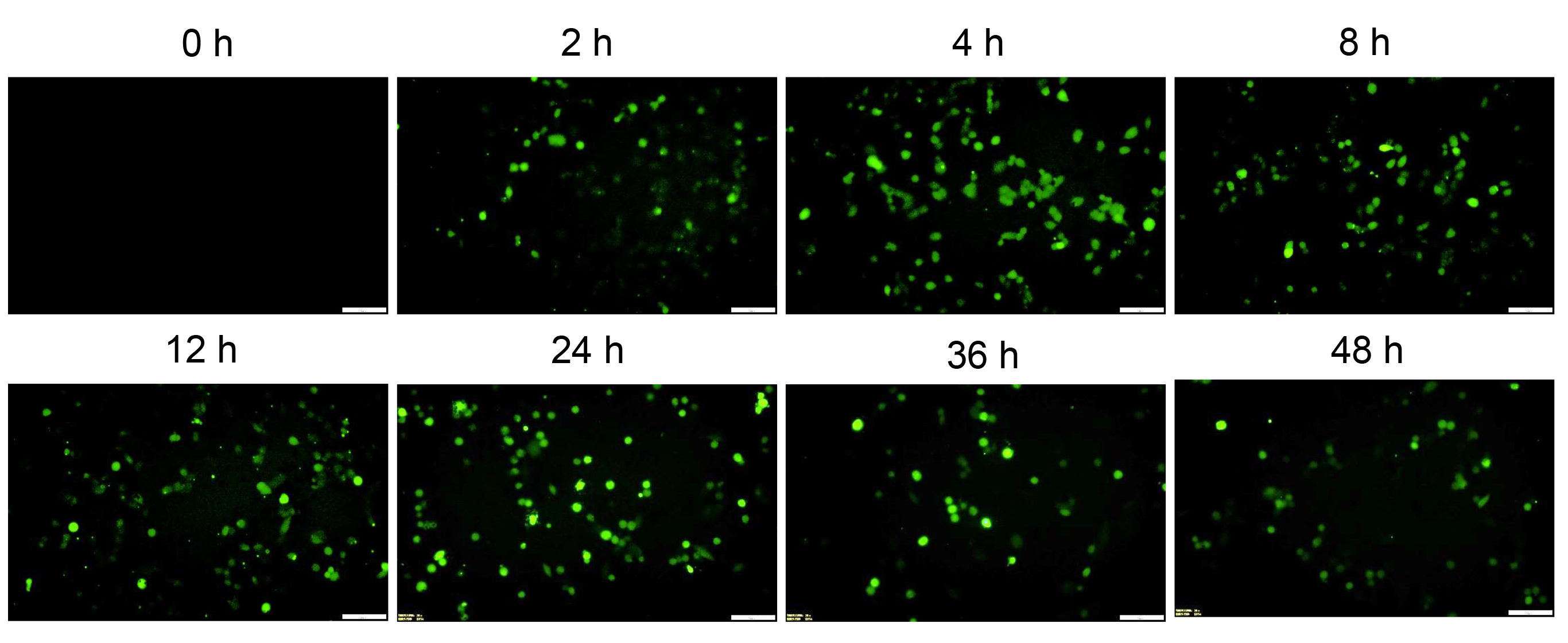


**Figure S23.** ROS production in A375 cells at different times after 8 μM Cu_6_NC treatment. Scale bar: 100 μm.


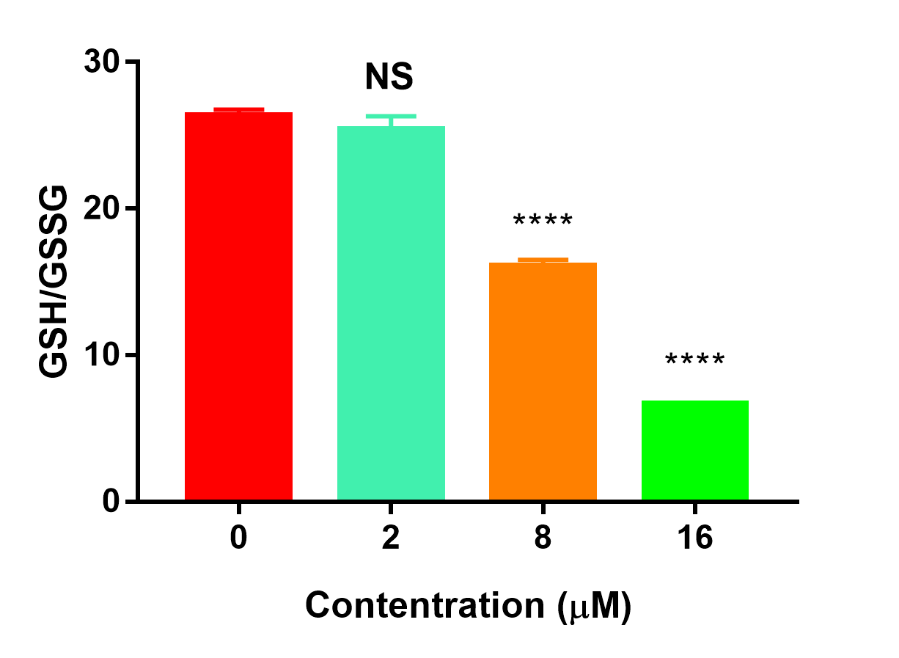


**Figure S24.** GSH/GSSG ratio in A375 cells after treatment different concentrations of Cu_6_NC.


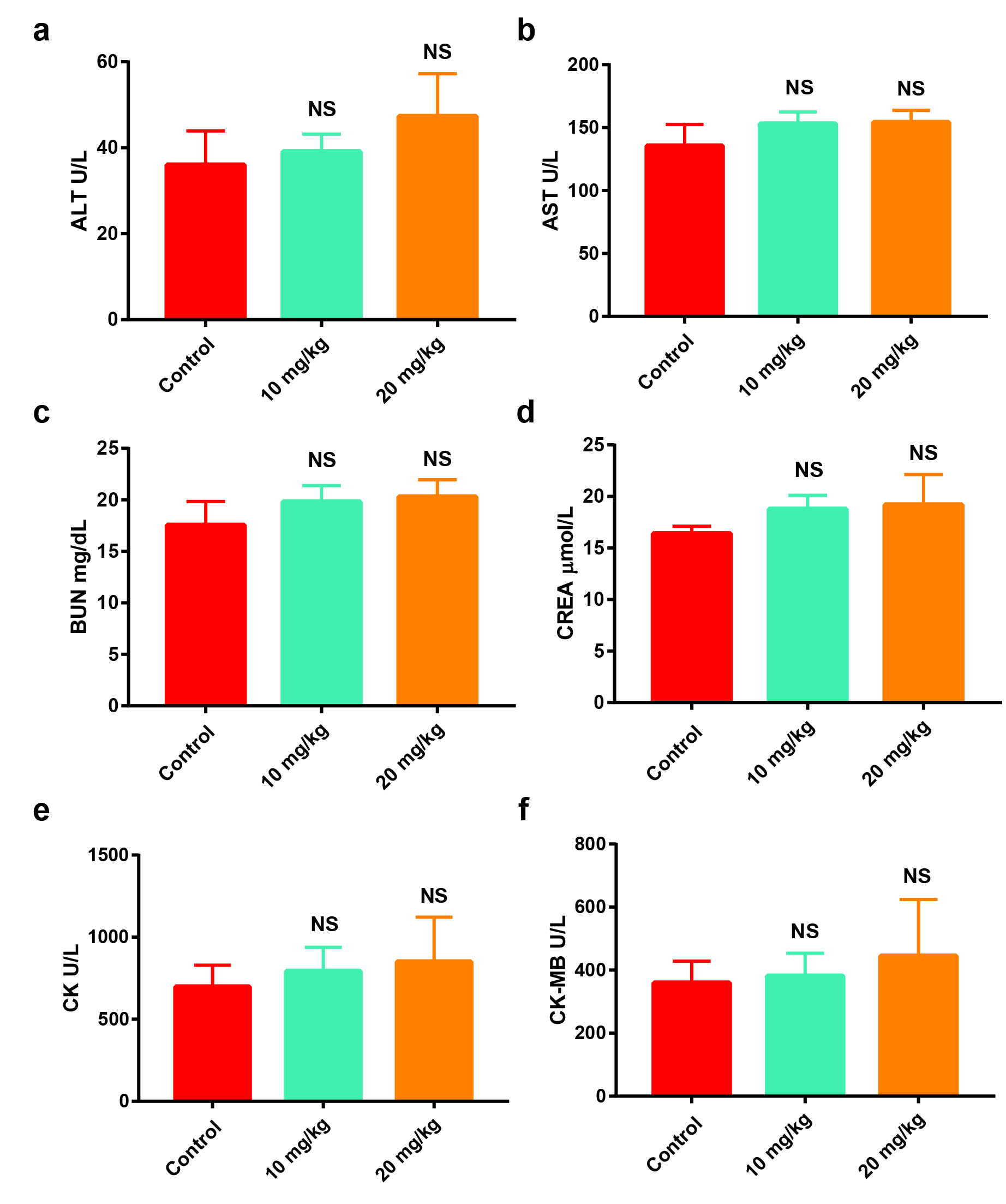


**Figure S25­­.** Quantitative analysis of blood parameters in mice injected with different concentrations of Cu_6_NC to assess the long-term toxicity. ALT, Alanine aminotransferase; AST, Aspartate aminotransferase; BUN, Blood urea nitrogen; CREA, Creatinine; CK, Creatine kinase; CK-MB, Creatine Kinase, MB Form.
